# Bulk RNAseq Analysis of Cardiac Myosin-Specific CD4^+^ and CD8^+^ T Cells Reveals Distinct Transcriptomic Profiles Between Myocarditis-Resistant and Susceptible Mice

**DOI:** 10.3390/biomedicines13112725

**Published:** 2025-11-06

**Authors:** Shraddha Singh, Meghna Sur, Kiruthiga Mone, Celia Wafa Ayad, Chandirasegran Massilamany, Arunakumar Gangaplara, Jay Reddy

**Affiliations:** 1 School of Veterinary Medicine and Biomedical Sciences, University of Nebraska-Lincoln, Lincoln, NE 68583, USA; ssingh22@huskers.unl.edu (S.S.); mesur@coh.org (M.S.); kmone2@huskers.unl.edu (K.M.); 2 Department of Immuno-Oncology, Beckmann Research Institute, City of Hope, Duarte, CA 91010, USA; 3 Center for Virology and Vaccine Research, Beth Israel Deaconess Medical Center, Harvard Medical School, Boston, MA 02215, USA; cayad@bidmc.harvard.edu; 4 Stealth-Mode Bio, Boston, MA 02494, USA; mchandirasegaran@gmail.com; 5 Miltenyi Biotec, Gaithersburg, MD 20878, USA; arungb@gmail.com

**Keywords:** Bulk RNAseq, transcriptomics, CD4^+^ T cells, CD8^+^ T cells, myocarditis, myocarditis susceptible, myocarditis resistant, myosin, C57BL/6, A/J, transcription factor

## Abstract

**Background**: We recently generated T cell receptor (TCR) transgenic (Tg) mice specific to cardiac myosin heavy chain-α (Myhc-α 334–352) on both myocarditis-resistant (C57BL/6) and susceptible (A/J) genetic backgrounds. We noted that the antigen-specific TCRs were expressed in CD4^+^ and CD8^+^ T cells in both strains, but their responses differed. While the T cells from naïve Tg C57BL/6 mice do not respond to Myhc-α 334–352, whereas those from A/J mice spontaneously respond to the antigen, suggesting their underlying molecular mechanisms might differ. **Methods**: To investigate the mechanisms of differences in the antigen-responsiveness between the Tg C57BL/6 and A/J mice, we performed bulk RNA sequencing on CD4⁺ and CD8⁺ T cells sorted by flow cytometry. Differentially expressed genes, Gene Ontology (GO), and Kyoto Encyclopedia of Genes and Genomes (KEGG) pathways, gene set enrichment analysis (GSEA) of GO and KEGG, and transcription factor (TF) network analyses were performed to identify pathways and regulators of immune responses. **Results**: First, the principal component analysis of the transcriptomic profiles distinguished CD4^+^ from CD8^+^ T cells, which also differed between the two strains. Second, the differentially expressed cytokine and cytotoxicity genes revealed similar patterns between CD4^+^ and CD8^+^ T cells. Importantly, KEGG enrichment analysis revealed downregulated pathways in both CD4^+^ and CD8^+^ T cells that are associated with viral myocarditis, and various autoimmune conditions in C57BL/6 as compared to A/J mice. Similarly, the GSEA of GO revealed negative regulation of heart contraction and positive regulation of cardiac muscle hypertrophy processes were negatively enriched in CD4^+^ T cells of C57BL/6 mice. Finally, by generating the transcription factor (TF) networks, 22 TFs were found common to both CD4^+^ and CD8^+^ T cells, whereas eight TFs were unique to CD4^+^ or CD8^+^ T cells that have a role in T cell activation, tolerance, and T regulatory cells. **Conclusions**: Our data provide new insights into the transcriptomic profiles that may contribute to the genetic resistance mechanisms for developing cardiac autoimmunity.

## 1. Introduction

One of the cardinal features of adaptive immune cells is non-reactivity to self-antigens in the periphery, and how this self-tolerance is broken, leading to pathogenic autoimmune responses, is a fundamental question in autoimmunity research. The maturation of lymphocytes in the thymus has been described based on affinity and stochastic selection models [1,2,3,4], where the affinity of peptide ligands to the T cell receptors (TCRs) determines the fate of T cells in the former [3,4], and the random termination of the expression of co-receptors (CD4^+^ or CD8^+^) influences T cell selection in the latter [3,4]. Consistent with the affinity selection model, the lymphocytes destined to be exported to the periphery are not expected to react strongly with the self-antigen complexed with the major histocompatibility complex (MHC) molecules. Conversely, lack of expression of self-antigens is also dangerous, as the self-reactive T cells escape negative selection, for which the transcription factors such as RUNX2, AIRE, and FEZ family zinc finger 2 are critical to the expression of tissue-specific antigens in the thymus [5,6,7]. This phenomenon has been verified with several self-antigens, including cardiac myosin heavy chain (Myhc)-α, by demonstrating the presence of Myhc-α-reactive T cells in the periphery that could be deleted upon expression of Myhc-α in the thymus [8]. Peripherally, the autoreactive T cells could remain tolerant, and various mechanisms such as clonal deletion, activation-induced cell death, lack of costimulatory molecules, and/or engagement with co-inhibitory molecules such as cytotoxic T lymphocyte-associated protein 4 and programmed cell death protein-1, and suppression by T regulatory (Treg) cells have been proposed [9,10,11,12]. While these mechanisms generally explain the maintenance of peripheral tolerance, clarity is needed on whether unique molecular pathways exist between genetically resistant and susceptible individuals. Most studies with T cell tolerance have focused on CD4^+^ T cells [13,14,15], and it is unclear whether the tolerance mechanisms of CD4^+^ and CD8^+^ T cells operate similarly or differently, and their delineation is critical.

We recently generated TCR transgenic (Tg) mice specific to cardiac Myhc-α 334–352 on both myocarditis-resistant, C57BL/6, and myocarditis-susceptible, A/J genetic backgrounds [16,17]. Functionally, although the antigen-specific TCRs were expressed in both CD4^+^ and CD8^+^ T cells, their antigenic responses differed. While the Tg T cells from naïve C57BL/6 mice did not respond to Myhc-α 334–352, the T cells from A/J mice spontaneously responded to antigen. However, immunization with Myhc-α 334–352 led us to note the responsiveness in both the CD4^+^ and CD8^+^ T cell subsets, suggesting that in vivo priming is necessary to break the tolerance in the resistance background [16]. Based on these observations, we tested the hypothesis that the molecular pathways regulating the unresponsiveness to antigen in C57BL/6 mice might differ by performing the bulk RNAseq analysis. The data revealed distinct transcriptomic profiles in CD4^+^ and CD8^+^ T cells that also differed between the two strains. While the Kyoto Encyclopedia of Genes and Genomes (KEGG) analysis revealed downregulation pathways, namely, viral myocarditis, and autoimmune conditions in both CD4^+^ and CD8^+^ Tg T cells of C57BL/6 mice, the gene sets important for heart contraction and cardiac muscle hypertrophy were negatively enriched, as analyzed by gene set enrichment analysis (GSEA) of Gene Ontology (GO) analysis. Likewise, the transcription factor (TF) analysis revealed important networks relevant to Treg cell functions, T cell tolerance, and autoimmunity. These processes may involve the roles of *Dapk1* and *Cxcr2* genes, which we noted consistently across DEGs, enrichment, and TF network analyses.

## 2. Materials and Methods

### 2.1. Mice

We previously reported the generation of TCR Tg mice specific to Myhc-α 334–352 [16,17]. In this study, we used Tg C57BL/6 mice and their backcrossed A/J mice (16–20 weeks) at generation four. The mice were genotyped by quantitative polymerase chain reaction (qPCR) as previously described [16]. All animals were maintained in accordance with the institutional guidelines of the University of Nebraska-Lincoln (protocol #2377), Lincoln, NE, USA. Animals were euthanized using a carbon dioxide chamber as recommended by the Panel on Euthanasia of the American Veterinary Medical Association.

### 2.2. Isolation of T lymphocytes and Sorting of CD4^+^ and CD8^+^ T Cells by Flow Cytometry

The peripheral lymph nodes and spleens were harvested from C57BL/6 and A/J Tg mice (*n* = 3), triturated, and filtered through a 70 µm cell strainer to obtain single-cell suspensions termed lymphocytes. The CD3^+^ T cells were enriched from the lymphocytes to a purity of ~100% by negative selection through magnetic separation using the MojoSort™ Mouse CD3^+^ T Cell Isolation Kit (BioLegend, San Diego, CA, USA). The enriched CD3^+^ T cells were incubated on ice for 15 min after staining with the following antibodies: anti-CD3^+^ (17A2), anti-CD4^+^ (GK1.5), anti-CD8^+^ (YTS156.7.7), Zombie Green viability dye (BioLegend), and anti-TCR Vβ4 (KT4) (BD Biosciences, Franklin Lakes, NJ, USA). The cells were then washed, resuspended in phosphate-buffered saline, and sorted by flow cytometry using CytoFLEX SRT (Beckman Coulter, Brea, CA, USA). Two distinct populations—Zombie^−^CD3⁺Vβ4⁺CD4⁺ (CD4^+^ T cells) and Zombie^−^CD3⁺Vβ4⁺CD8⁺ T cells (CD8^+^ T cells) —were sorted and stored in QIAzol lysis reagent (Qiagen, Hilden, Germany) at −80 °C until further analyses.

### 2.3. RNA Isolation and Bulk RNA Sequencing

Total RNA was extracted using the RNeasy Plus Universal Mini Kit (Qiagen, Hilden, Germany) and eluted in RNase-free water according to the manufacturer’s recommendations. The purity and concentration of the RNA were quantified using a NanoDrop One spectrophotometer (Thermo Scientific, Waltham, MA, USA) and a Qubit 4 Fluorometer (Thermo Fisher Scientific, Waltham, MA, USA). A total of 100 ng RNA samples were sent to Novogene for sequencing (Sacramento, CA, USA). RNAseq was performed using the Illumina platform based on sequencing by synthesis [18]. In brief, messenger RNA was purified from total RNA using poly-T-oligo-attached magnetic beads, and the first-strand cDNA was synthesized using random hexamers; the second-strand cDNA was synthesized using dUTP for a directional library or dTTP for a non-directional library. The libraries were checked using Qubit, real-time PCR for quantification, and a bioanalyzer for size distribution. The qualified libraries were then sequenced for further analysis.

### 2.4. Transcriptomic Analysis

The quality of the sequencing raw data was checked using FastQC (version 0.12.0) [19]. To ensure data reliability, raw reads were filtered to remove low-quality sequences and adapter contaminants to obtain high-quality clean reads for further analysis. The filtered reads were mapped to the mouse genome using HISAT2 (version 2.2.1) [20]. Gene expression levels were analyzed based on the abundance of sequencing reads mapped to the genome or exonic regions [21] and gene expression was normalized and expressed as fragments per kilobase of transcript per million mapped reads (FPKM), which adjusts read counts for both gene length and sequencing depth, providing a standardized measure of gene expression [22]. The quantification of gene expression was performed by FeatureCounts (version 2.0.6) [23].

### 2.5. Differentially Expressed Genes (DEGs) Analysis

DEG analysis was performed to identify significantly altered genes in CD4^+^ and CD8^+^ T cells between resistant (C57BL/6) and susceptible (A/J) Tg mice. Using gene expression quantifications, the genes exhibiting significant differences in their expression between experimental groups were identified. The differential analysis was then performed using the DESeq2 package (version 1.42.0), with an adjusted P-value (Padj) cutoff of 0.05 using the Benjamini–Hochberg adjustment method [24].

### 2.6. Enrichment Analysis

Enrichment analyses were performed to analyze the biological processes, metabolic, and disease pathways in the DEGs. Both GO and KEGG analyses for up- and downregulated genes were performed using clusterProfiler (version 4.8.1). The enriched pathways with Padj value < 0.05 were considered statistically significant. To identify enriched pathways, we performed ranked gene list analysis on the whole gene expression level using GSEA software (version v4.3.2). We next performed GSEA of GO and KEGG analyses to identify various processes and pathways using the overall gene sets. GSEA evaluates a ranked list of genes by their differential expression metrics to calculate an enrichment score (ES) for each pathway. The ES increases when genes within a given pathway appear at the top of the ranked list and decreases when they are absent or appear further down the list. Consequently, pathways enriched with highly ranked genes exhibit amplified ES values, while those with moderately ranked genes show minimal enrichment. The normalized enrichment score (NES) represents the degree of pathway enrichment, calculated as the maximum deviation of the running sum statistic normalized to the pathway size. A positive NES indicates enrichment of genes at the top of the ranked list, whereas a negative NES reflects enrichment at the bottom. An NES ≥1.5 and a false discovery rate (FDR) q-value ≤ 0.25 were considered statistically significant in the enrichment plots.

### 2.7. Real-Time qPCR

To validate a subset of DEGs identified by RNA sequencing, qPCR analysis was performed. Total RNA used for qPCR was derived from the same aliquots of RNA samples previously used for RNA-seq. The RNA samples were diluted to a final concentration of 12.5 ng/µL in nuclease-free water before amplification. The qPCR reactions were carried out using the iTaq™ Universal SYBR^®^ Green One-Step Kit (Bio-Rad, Hercules, CA, USA) on a CFX96™ Real-Time PCR Detection System (Bio-Rad). Each 20 µL reaction contained 2 µL of RNA (12.5 ng/µL), iTaq Universal SYBR Green reaction mix, and 10 pM gene-specific primers. The primer pairs in the order of forward and reverse sequences include: Bcl-2 modifying factor (*Bmf*), AAAATGGAGCCACCTCAGTGTGTG and TCACCAGGGCCCCACCCCTTC; cytochrome P450 family 2 subfamily r member 1 (*Cyp2r1*), CA CGTCTACATGAGGAAGCAGAG and GGAAGGCATGGTCTATCTGC; interleukin 12 receptor subunit beta 2 (*Il12rb2*), GTGGGGTGGAGATCTCAGTTG and CCAGAGTTCCAGGAACAG; granzyme K (*GzmK*), TGTCCAACTGCTTCACCTGGG and GCCACCAGAGTCACCCTTGCA; and C-X-C motif chemokine receptor 2 (*Cxcr2*), CTTTCTTCCAGTTCAACCAGC and TCCACCTTGAATTCTCCCATC. Gene expression levels were normalized to the housekeeping gene glyceraldehyde-3-phosphate dehydrogenase (*Gapdh*), CTCCCACTCTTCCACCTTCG, and GCCTCTCTTGCTCAGTGTCC. Reactions were performed in duplicates, and quantification was based on the comparison of the cycle threshold (Ct) values of the target genes relative to those of *Gapdh*. The ΔCt value was calculated as the difference between the Ct of the gene of interest and the Ct of *Gapdh*. Expression of target genes was normalized to *Gapdh* using the 2^−(ΔΔCt)^ method [25,26].

### 2.8. TF Network Analysis

We performed TF network analysis to identify regulatory interactions using DEGs. The gene expression data were filtered to retain sufficiently expressed genes by filtering out genes with raw counts <10, and the gene regulatory networks (GRNs) were constructed by integrating TF-target interactions. These interactions were obtained by combining three datasets: TRRUST [27], RegNetwork [28], and DoRothEA [29]. The DEGs were identified and mapped onto the GRNs to generate DEG-centered subnetworks that included their upstream regulators. TFs were inferred based on network connectivity, and the most highly connected TFs were highlighted as master regulators. Regulatory relationships of selected TFs were further characterized by extracting their top-ranked target genes, and subnetworks were refined to retain only significant regulators and targets. Two complementary visualizations were generated: (a) a global overview highlighting the top master regulators, and (b) focused subnetworks of selected TFs and their regulated genes. All analyses were performed in R using the following packages: dplyr for data manipulation and igraph for graph construction and network analysis. For visualization, graphs were converted with tidygraph and plotted using ggraph for a high-resolution PNG export of transcription factor networks with annotated node and edge attributes.

### 2.9. Statistical Analysis

Comparison between the groups with respect to qPCR analysis was performed by Student’s t-test [30]. The graphs were generated using GraphPad Prism software v8.0 (GraphPad Software, Inc., La Jolla, CA, USA). The *p*-values ≤ 0.05 were considered significant.

## 3. Results and Discussion

We recently reported the generation of TCR Tg mice specific to Myhc-α 334–352 on both myocarditis-resistant C57BL/6 and myocarditis-susceptible A/J [16,17] genetic backgrounds. But their responses differed in that the naïve Tg T cells from A/J but not C57BL/6 mice responded to antigen [16], leading us to perform transcriptomic analysis to identify the genes that could potentially contribute to the maintenance of T cell tolerance in these mice.

### 3.1. Expression of Transcriptomic Profiles Differs between C57BL/6 and A/J Mice in CD4^+^ and CD8^+^ T Cells

**Figure 1:**
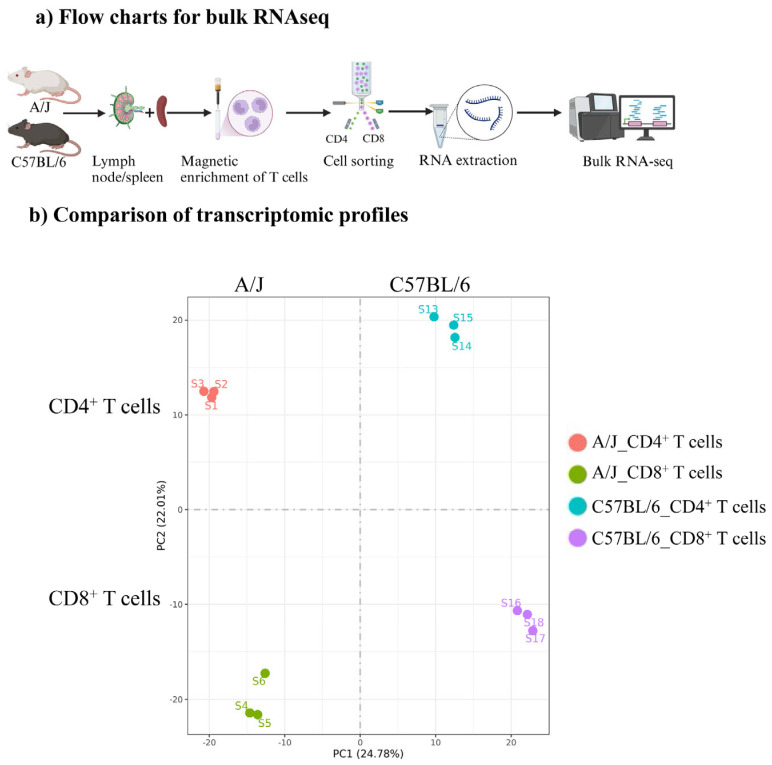
Bulk RNAseq analysis of CD4^+^ and CD8^+^ T cells from Tg C57BL/6 and A/J mice. (**a**) Flow charts for bulk RNAseq. Schematic representation of the methodology employed for bulk RNAseq. The CD3^+^ T cells enriched by negative selection from C57BL/6 and A/J mice (*n* = 3) were flow cytometrically sorted to purify CD4^+^ and CD8^+^ T cells. The RNA samples corresponding to each subset were subjected to bulk RNAseq. (**b**) Comparison of transcriptomic profiles. Distribution of CD4^+^ and CD8^+^ Tg T cells from C57BL/6 and A/J mice (*n* = 3). The PCA plot was generated using the gene expression value quantified as FPKM from individual samples to visualize the variance and clustering patterns between the two mouse strains and T cell subtypes.

We performed bulk RNAseq analysis to understand the differences in the antigenic responsiveness of T cells between C57BL/6 and A/J Tg mice. Essentially, CD4^+^ and CD8^+^ T cells were sorted to ~100% purity using the single-cell suspensions obtained from the spleens and lymph nodes of naïve Tg mice (Supplementary Figures S1 and S2). RNA extracted from each subset was subjected to bulk RNAseq analysis, and quantification of gene expression was analyzed by FeatureCounts software (version 2.0.6) (Figure 1a) [23]. By using the FPKM values derived for each sample in a group, the principal component analysis (PCA) plot was generated, which facilitated the visualization of variance and clustering between CD4^+^ and CD8^+^ T cells and also between groups (C57BL/6 vs. A/J). The PCA plot revealed a striking difference in the segregation of transcripts between the two strains and the gene expression patterns of biological replicates within CD4^+^ or CD8^+^ T cell subsets in each strain, aligned distinctly (Figure 1b). Furthermore, we noted a clear segregation of transcriptomic profiles in both T cell subsets. Of note, CD4^+^ T cells, being the helper T cells, mediate their functions by producing cytokines, as opposed to the cytotoxic functionality of CD8^+^ T cells [31,32,33]. Thus, the distinct transcriptomic profiles noted in these subsets may suggest a potential role in their corresponding functions that could be influenced by the genetic backgrounds in the maintenance or break in T cell tolerance.

### 3.2. DEG Analysis Revealed Overlapping Transcripts between CD4^+^ and CD8^+^ T Cells, but Their Expression Differed

**Figure 2:**
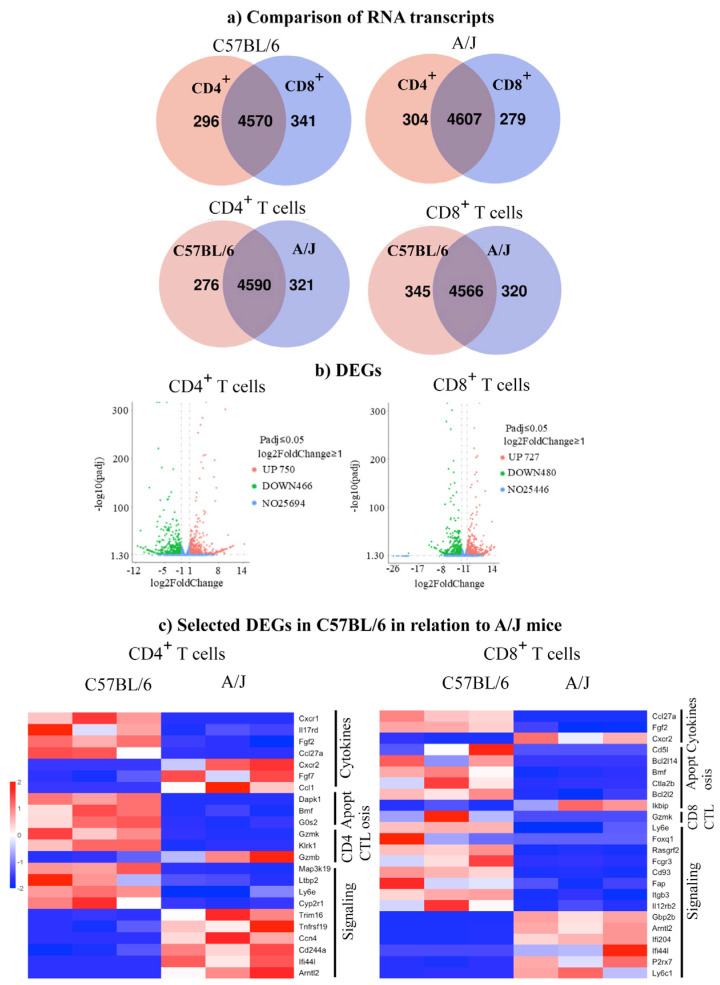
RNAseq analysis in the Tg C57BL/6 and A/J mice reveals distinct gene expression patterns in CD4^+^ and CD8^+^ T cells. (**a**) Comparison of RNA transcripts. Distribution of uniquely expressed and overlapped genes in CD4^+^ and CD8^+^ T cell populations sorted from C57BL/6 and A/J mice (*n* = 3) using a cutoff of 10 for expressed genes. (**b**) DEGs. Volcano plots showing differentially expressed genes in CD4^+^ and CD8^+^ T cells from C57BL/6 and A/J mice (*n* = 3). The plots display genes that are significantly differentially expressed based on the Padj threshold of < 0.05 and a log_2_ fold change > 1. The upregulated genes are indicated in red dots, while downregulated genes are shown in green dots. (**c**) Selected DEGs in C57BL/6 in relation to A/J. The DEGs associated with immune response were identified in CD4^+^ and CD8^+^ T cells based on the Padj threshold of 0.05 and a log_2_ fold change >2. Upregulated genes are shown in red; in contrast, downregulated genes are shown in blue.

**Figure 3:**
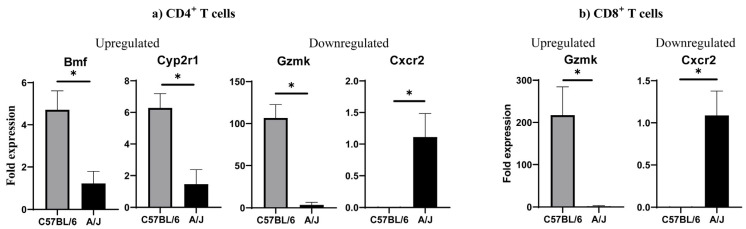
Validation of selected DEGs by qPCR in CD4^+^ (**a**) and CD8^+^ T (**b**) cells from Tg mice. CD4+ and CD8+ T cells were sorted flow cytometrically from Tg mice. Total RNA was extracted, and qPCR was performed to assess the expression of selected genes in each T cell subset (panel a, CD4^+^ T cells; panel b, CD8^+^ T cells). Gene expression levels were normalized to GAPDH, and relative expressions were calculated using the 2^−(∆∆Ct)^ method. Data are presented as mean ± SEM, with *n* = 3 mice per group. Statistical significance was determined using a Student’s t-test; * *p* < 0.05 was considered significant.

We performed DEG analysis to gain insights into the maintenance of tolerance in C57BL/6 mice by comparing their transcript profiles with A/J mice. First, as shown in the Venn diagram, we noted the expression of 296 and 341 transcripts, respectively, in CD4^+^ and CD8^+^ T cells from C57BL/6 mice with an overlap of 4570 transcripts between the two (Figure 2a). Similar analysis revealed 304 and 279 transcripts in the corresponding subsets, in addition to 4607 transcripts in A/J mice. Further analysis within the CD4^+^ subset indicated 276 and 321 transcripts unique to C57BL/6 and A/J mice, whereas 4590 transcripts were found overlapping between the two strains (Figure 2a). Likewise, within the CD8^+^ T cell subset, 345 and 320 transcripts were unique to C57BL/6 and A/J mice, with 4566 overlapping transcripts (Figure 2a). These observations suggest that the uniquely expressed transcripts may have different functions. To that end, we sought to identify the up- and downregulated genes using log2-fold change more than 1, and a Padj threshold of less than 0.05. We compared the transcripts in CD4^+^ or CD8^+^ T cell subsets, as shown in the volcano plots (Figure 2b). The analyses revealed 750 upregulated and 466 downregulated genes in the CD4^+^ T cell subsets, whereas in the CD8^+^ T cell subset, 727 and 480 transcripts were found up- and downregulated, respectively (Figure 2b). Next, we used log2-fold change more than 2 and a Padj threshold of less than 0.05, which allowed us to group genes relevant to various immune functions, including cytokine activity, apoptosis, cytotoxicity, and signaling. Within the cytokine group in CD4^+^ T cells, we noted upregulation of *Cxcr1, Ccl27a, Il17rd*, and *Fgf2*, whereas *Fgf7, Cxcr2*, and *Ccl1* were downregulated in C57BL/6 mice (Figure 2c, left panel, and Supplementary Tables S1 and S2). Similarly, three transcripts (*Dapk1, Bmf*, and *Gos2*) related to apoptosis were upregulated. However, variable expressions of transcripts having a role in cytotoxicity (upregulated, *GzmK* and *Klrk1*; and downregulated, *GzmB*) were noted in the CD4^+^ T cell subset. As to signaling molecules, four transcripts (*Ly6e, Map3k19, Cyp2r1*, and *Ltbp2*) were found upregulated, in addition to downregulated genes (*Ifi44l, Cd244a, Trim16, Ccn4, Tnfrsf19*, and *Arntl2*). By performing similar analysis for CD8^+^ T cells, the patterns of cytokines and their receptors indicated above resembled those of CD4^+^ T cells, except that the expression of *Cxcr1, Il17rd,* and *Ccl1* was lacking in the CD8^+^ T cell subset (Figure 2c right panel) (Supplementary Table S3 and S4). Regarding apoptotic genes, except *Bmf*, which is common to both CD4^+^ and CD8^+^ T cells, four genes were upregulated (*Bcl2114, Bcl212, Ctla2b*, and *Cd5l*), whereas *Ikbip* was downregulated in the CD8^+^ T cells. However, *GzmK* expression was upregulated in CD8^+^ T cells, similar to CD4^+^ T cells. Additionally, eight transcripts relevant to signaling molecules were found upregulated (*Ly6e, Foxq1, Rasgrf2, Fcgr3, Cd93, Fap, Itgb3*, and *Il12rb2*), whereas five others (*Ly6c1, Gbp2, Arntl2, Ifi204*, and *Ifi44l*) were downregulated in CD8^+^ T cells. The patterns of three of these (*Ly6e, Arntl2*, and *Ifi44l*) were, however, similar to those of CD4^+^ T cells. We then validated a few selected DEGs by qPCR analysis. Expectedly, the upregulated genes analyzed by bulk RNAseq were also found to follow a similar pattern as noted in CD4^+^ T cells (upregulated, *Bmf, Cyp2r1*, and *GzmK*, and downregulated, *Cxcr2*) (Figure 3a and Supplementary Figure S3) and CD8^+^ T cells (upregulated, *GzmK,* and downregulated, *Cxcr2*) (*p* < 0.05) (Figure 3b and Supplementary Figure S3). Overall, the DEG analysis revealed alterations in several genes common to both CD4+ and CD8^+^ T cells, suggesting similar functions in both subsets, whereas the genes expressed uniquely in CD4^+^ or CD8^+^ T cells may have functional relevance to each subset. For example, the upregulated gene, *GzmK*, related to cytotoxicity may be functionally relevant to both subsets [34]. Similarly, the downregulated *Cxcr2*, which facilitates leukocyte migration, may be critical for inducing cardiac autoimmunity, as demonstrated in the experimental autoimmune encephalomyelitis (EAE) model [35].

### 3.3. GO and KEGG Enrichment Analyses Revealed Biological and Metabolic Pathways Common to CD4^+^ and CD8^+^ T Cells

**Figure 4:**
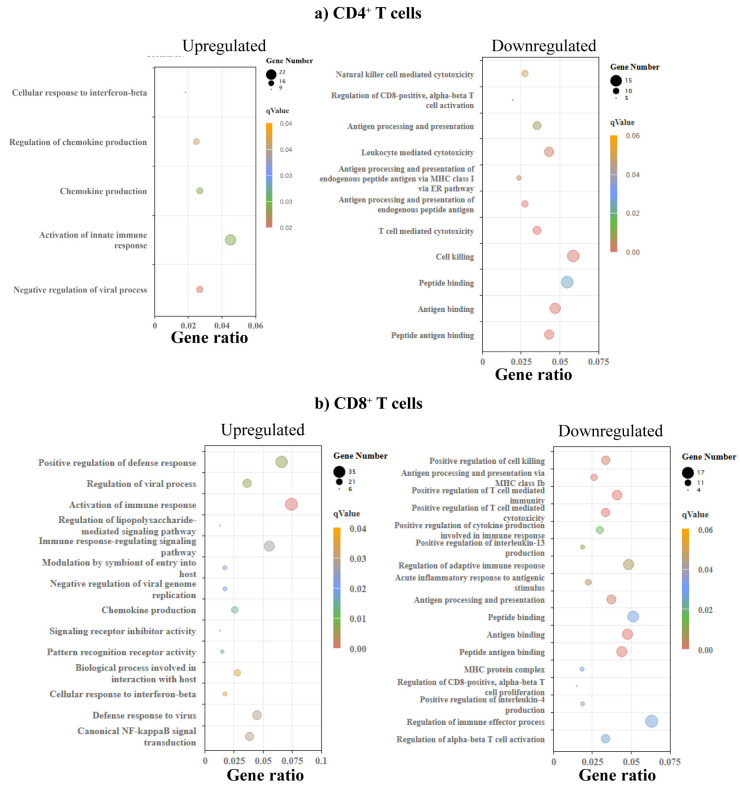
GO enrichment analysis revealed varied biological processes in CD4^+^ and CD8^+^ T cells. (**a**) CD4⁺ T cells. The left panel shows terms enriched among upregulated genes, and the right panel shows terms enriched among downregulated genes in C57BL/6 relation to A/J mice (*n* = 3). (**b**) CD8⁺ T cells. The left and right panels show terms enriched in upregulated and downregulated genes. The size of a point represents the number of genes annotated to a specific GO term, and the color represents the significance level of the enrichment.

To determine the significance of DEGs, we sought to identify the biological processes based on the GO enrichment analysis. We noted upregulation of pathways of chemokine production and their regulation, and cellular response to interferon (IFN)-β in CD4^+^ T cells (Figure 4a, left panel and Supplementary Table S5). Likewise, pathways relevant to T cell/leukocyte-mediated cytotoxicity were downregulated (Figure 4a, right panel and Supplementary Table S5). Similar analysis for CD8^+^ T cells resulted in the identification of upregulation of canonical NF-κB signal transduction and signaling receptor inhibitor activity, in addition to cellular response to IFN-β and chemokine production (Figure 4b, left panel and Supplementary Table S5). The downregulated processes in CD8^+^ T cells include T cell-mediated cytotoxicity, regulation of CD8 T cell proliferation, and positive regulation of cytokines such as interleukin (IL)-13 and IL-4 (Figure 4b, right panel and Supplementary Table S5). By comparing these profiles between CD4^+^ and CD8^+^ T cells, we noted common pathways both in the upregulated (chemokine production and response to IFN-β) and downregulated (T cell-mediated cytotoxicity) categories. A number of other downregulated pathways related to antigen processing or peptide binding were also noted (Supplementary Table S5).

We next asked whether the genes common to both CD4^+^ and CD8^+^ T cells contribute to different enrichment pathways, leading us to note two transcripts, namely, *Lilrb4* and *Fosl2*, as potential candidates (Supplementary Table S5). Expression of *Lilrb4*, including *Lilrb5*, has been reported to mediate upregulation of immune suppressive cytokine, IL-10, and downregulation of a chemokine, IL-8, that promotes leukocyte migration [36]. Conversely, *Fosl2* could promote autoimmunity by repressing Treg development, and reports indicate that the mice overexpressing *Fosl2* develop a systemic inflammatory phenotype, whereas *Fosl2*-deficient mice show reduced severity of EAE [37]. Further, we noted three transcripts*, Crtam, Klrb1*, and *Cd1d* of critical importance to T cell-mediated cytotoxicity in both CD4^+^ and CD8^+^ T cell subsets (Supplementary Table S5). *Crtam* is a member of the immunoglobulin superfamily, and its expression has been reported in the activated cytotoxic functionalities of CD4^+^, CD8^+^, and NK T cells [38,39], whereas *Klrb1* signifies activated CD4^+^ T cells in Sjögren’s syndrome [40]. While both *Crtam* and *Klrb1* could promote autoimmunity [38,39,40], failure to upregulate *Cd1d* could lead to the generation of Treg cells [41]. We noted *GzmB* in CD4^+^ T cells, *Nlrp3, P2rx7*, and *Slamf1* in CD8^+^ T cells that may influence cytotoxicity and cytokine production pathways in the respective subsets (Supplementary Table S5). While *GzmB* is a well-known marker of cytotoxicity [42], the effects of which can also be influenced by *P2rx7* [43], *Nlrp3* is involved in inflammatory cytokine production [44], and *Slamf1* inhibits T and B cell interaction and diminishes IL-6 production [45]. These observations suggest that alterations in the expression of genes may occur simultaneously, but the skewed expression of a set of genes might influence individual pathways.

**Figure 5:**
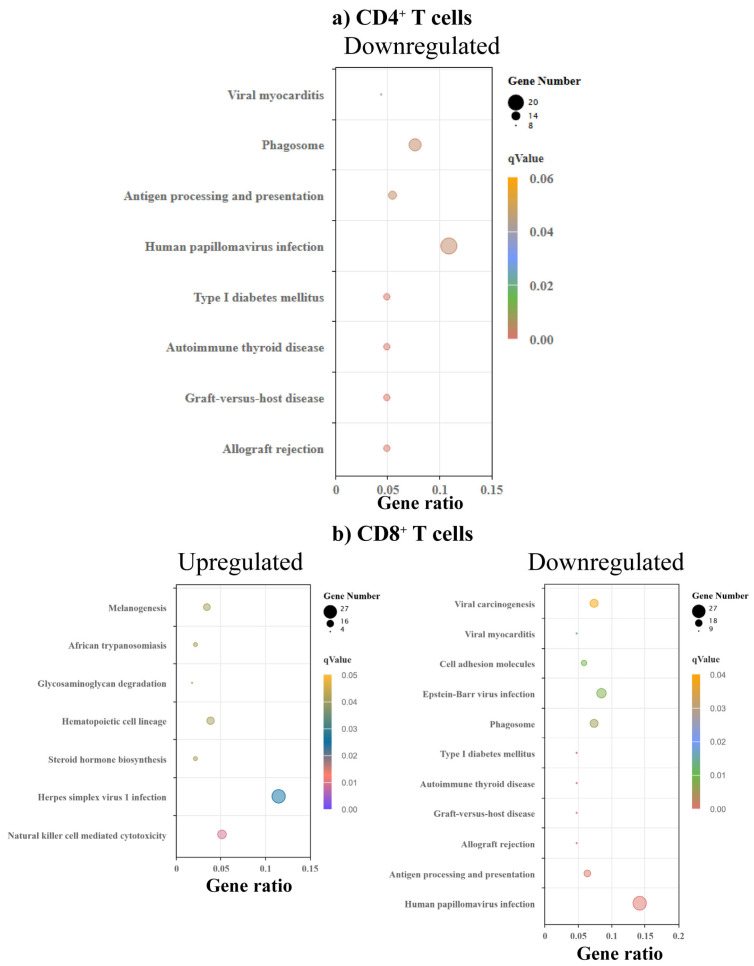
KEGG enrichment analysis revealed varied pathways associated with various disease conditions and metabolic pathways in CD4^+^ and CD8^+^ T cells. (**a**) CD4^+^ T cells. Bulk RNA-seq analysis of KEGG enrichment of DEGs in CD4^+^ T cells from C57BL/6 mice compared to A/J mice (*n* = 3). The top panel displays enriched KEGG pathways associated with downregulated genes. (**b**) CD8^+^ T cells. KEGG enrichment of DEGs in CD8^+^ T cells from C57BL/6 mice compared to A/J mice (*n* = 3). The left and right panels show pathways enriched in upregulated and downregulated genes, respectively. The size of a point represents the number of genes annotated to a specific KEGG pathway. The color represents the significance level of enrichment.

To determine the metabolic pathways and potential disease associations with the differentially altered genes, we performed KEGG enrichment analysis. While we noted no significantly upregulated pathways in CD4^+^ T cells, four pathways (type 1 diabetes, T1D, autoimmune thyroid disease, ATD, graft-versus-host disease, GVHD, and allograft rejection) were downregulated, in addition to viral myocarditis, antigen processing, and papilloma virus infection (Figure 5a and Supplementary Table S6). Similar analysis in CD8^+^ T cells indicated seven upregulated pathways, of which African trypanosomiasis, and herpes simplex virus (HSV)-1 infection are linked to myocardial disease (Figure 5b) [46,47,48]. Similarly to CD4^+^ T cells, we noted downregulation of the autoimmune (T1D and ATD) and alloreactivity pathways (GVHD and allograft rejection besides viral myocarditis, antigen processing and papilloma virus infection, and Epstein–Barr virus (EBV) in CD8^+^ T cells (Figure 5b and Supplementary Table S6). Reports indicate that African trypanosomiasis and HSV-1 infection are linked to heart disease, but the significance of upregulation of these pathways only in CD8^+^ T cells is unclear. On the other hand, downregulation of autoimmune and alloreactivity pathways in both CD4^+^ and CD8^+^ T cells could be relevant to myocarditis-resistance in C57BL/6 mice. This proposition can be further supported by the downregulation of pathways related to papilloma virus and EBV infections, which increase the risk of heart disease and myocarditis, respectively [49,50,51]. We also noted a set of non-classical MHC genes, namely, *H2-Q1, H2-T3, H2-B1*, and *H2-Ea*, that were found to be associated with the alterations in the autoimmune and alloreactivity pathways, and viral myocarditis, as indicated above in both CD4^+^ and CD8^+^ T cells (Supplementary Table S6). Since these pathways were downregulated in myocarditis-resistant C57BL/6 mice, the data suggest that the gene loci encompassing *H2-Q1, H2-T3, H2-B1*, and *H2-Ea* genes might influence their disease-resistant phenotype. In support of this proposition, nonclassical MHC genes such as HLA-G and HLA-E have been linked with autoimmune diseases [52,53,54].

### 3.4. GSEA-Enrichment Analyses Revealed Distinct Pathways in CD4^+^ and CD8^+^ T Cells

**Figure 6:**
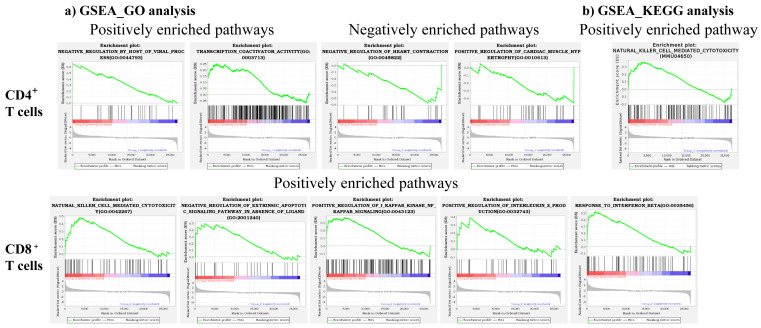
GSEA reveals differentially enriched GO and KEGG pathways in CD4⁺ and CD8⁺ T cells. (**a**) GSEA_GO analysis. GSEA of GO term enrichment revealed both positively and negatively enriched pathways in CD4^+^ and CD8^+^ T cells in C57BL/6 compared to A/J mice (*n* = 3). (**b**) GSEA_KEGG analysis. GSEA of KEGG revealed positively enriched pathways in CD4^+^ T cells in C57BL/6 compared to A/J mice (*n* = 3). In each GSEA plot, the top portion shows the enrichment score, representing the degree of overrepresentation of the gene set across the ranked list. Red indicates upregulated genes and blue indicates downregulated genes. The vertical black lines indicate the positions of genes from the selected gene set within this ranked list. The bottom portion of the plot illustrates the rank metric used to order genes. Enrichment plots represent NES, with significance determined using an FDR q-value < 0.25 and a NES > 1.5.

We performed GSEA of GO and KEGG analysis to identify variations in different biological processes, metabolic, and disease pathways. These analyses involved the comparison of the consideration of all gene sets expressed in either CD4^+^ or CD8^+^ T cells in C57BL/6 mice in relation to A/J mice. From the top 50 pathways representing the GO and KEGG analyses in both CD4^+^ and CD8^+^ T cells, we selected seven pathways (GO, nine, and KEGG, one) using two criteria, namely, NES more than 1.5 and FDR q-values less than 0.25 [55], where the selected gene candidates are indicated in parentheses. The GSEA enriched GO plots involved the transcription activator activity pathway (*Fgf2*) in the positively enriched processes of the CD4^+^ T cell subset that has been previously shown to be important for myocardial protection (Figure 6a and Table 1) [56]. In contrast, two processes, negative regulation of heart contraction (*Tnf, Pde5a*, and *Trpv1*) and positive regulation of cardiac muscle hypertrophy (*Pin1* and *Ccn4*), were noted in the negatively enriched category (Figure 6a and Table 1). The findings that *Tnf* is critical for the survival of autoreactive T cells [57], the association of increased cardiovascular risk with *Pde5* [58], *Trpv1* and *Pin1* with autoimmunity [59,60,61], and *Ccn4* with signaling pathways [62], suggest that these genes may indirectly contribute to the regulation of heart contractile functions. Similar analysis in the positively enriched gene sets of CD8^+^ T cells revealed three processes related to NK cell-mediated cytotoxicity (*Klr*s and *GzmB*), positive regulation of IkB signaling of NF-κB pathway (*Tnf* and *Fasl*), and IL-2 production (*Tnf, Il-1a*, and *IL-1b*) (Figure 6a and Table 1). While the enrichment of *Klrs*, *GzmB*, and *Tnf* was expected in CD8^+^ T cells because of their involvement in cytotoxicity [42,63,64], upregulation of *Fasl* may be a key contributing factor since the Fas-Fasl pathway is critical to control the expansion of autoreactive T cells [65]. The KEGG analysis also revealed genes (*Klr*s) related to the NK cell-mediated cytotoxicity pathway in the positively enriched CD4+ T cell subset (Figure 6b and Table 1), and other pathways were found not relevant to T cell functions. Taken together, the finding that the cytotoxicity genes, especially *Klr*s, were enriched in GSEA of GO and KEGG analysis in both CD4^+^ and CD8^+^ T cells suggests that both cell types could mediate cytotoxicity function. Our recent data demonstrating the expression of various cytotoxic markers common to both CD4^+^ and CD8^+^ T cells supports this proposition [66].

### 3.5. TF Network Analysis Revealed Regulatory Circuits of Importance to T cell Responses

**Figure 7:**
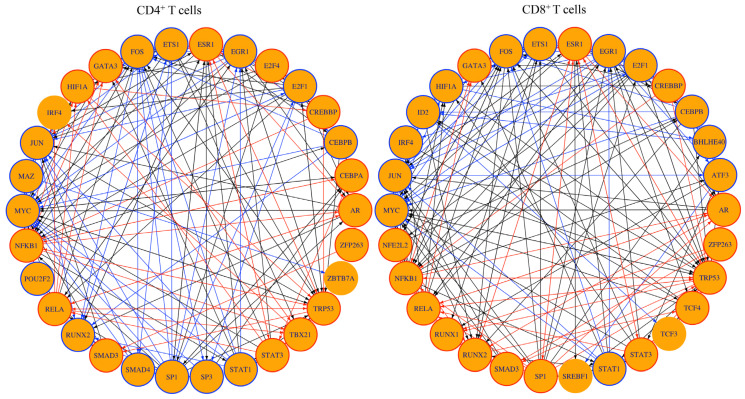
Transcription factor network analysis revealed TFs common to both CD4^+^ and CD8^+^ T cells in addition to a set of TFs unique to each subset. Transcription factor network analysis of DEGs in CD4^+^ (left panel) and CD8^+^ T cells (right panel) from C57BL/6 in comparison to A/J Tg mice (*n* = 3) using a Padj value cut off <0.05 and a log_2_ fold change ≥2. Shown are the 30 transcription factors with the highest downstream regulation effects. The resulting master regulator network is visualized as a circular layout, and the TF-target subnetwork is visualized in a tree layout. Orange nodes represent transcription factors, and blue nodes represent regulated genes that are not transcription factors. Directed edges indicate interactions or regulatory relationships. Red node borders link upregulated genes to their corresponding master regulators, while blue edges connect downregulated genes to their predicted regulators. Black edges indicate a mixed regulation, meaning that one node is upregulated and the other is downregulated.

To determine whether the genes expressed in CD4^+^ and CD8^+^ T cells could be regulated by TFs unique to each subset, we performed the TF network analysis. For TF network analysis, we filtered the transcriptomic profiles based on two criteria (Padj value ≤ 0.05 and log 2-fold change ≥2), leading us to select the top 30 TFs in CD4^+^ or CD8^+^ T cells (Figure 7). Of these, 22 TFs were common to both subsets, in which 10 TFs were linked with the upregulated genes (ESR1, CREBBP, AR, ZFP263, TRP3, STAT3, SMAD3, RELA, NFKB1, GATA3), and nine TFs were linked with downregulated genes (EGR1, E2F1, CEBPB, STAT1, MYC, JUN, IRF4, FOS, and ETS1) in both subsets (Supplementary Table S7). Notably, four of the TFs linked to upregulated genes, namely CREBBP, TRP3, SMAD3, and GATA3, have been reported for the induction of Treg cells by directly modulating the expression of FoxP3 [67,68] or indirectly for the maintenance of Treg cells independent of modulation of FoxP3 expression [69,70]. Likewise, STAT3, RELA, and NFKB1 could contribute to peripheral T cell tolerance that may also modulate Treg cell functions, as noted with RELA and NFKB1 [71,72,73,74]. Three TFs, namely, STAT1, MYC, and FOS-JUN, promoting autoimmunity or T cell activation [75,76,77] were associated with the downregulated genes (Supplementary Table S7). Further analysis revealed eight TFs to be unique to CD4^+^ T cells (E2F4, CEBPA, ZBTB7a, TBX21, SP3, SMAD4, and POU2F2) or CD8^+^ T cells (BHLHE40, ATF3, TCF4, TCF3, SREBF1, RUNX1, NFE2L2, and ID2), whereas three TFs, HIF1a, SP1, and RUNX2 differed in their patterns (Supplementary Table S7). In relation to the CD4^+^ T cell subset, the TFs E2F4, CEBPA, TBX21, and HIF1a were associated with upregulated genes, as opposed to ZBTB7a, SP3, SMAD4, POU2F2, MAZ, SP1, and RUNX2, which were associated with downregulated genes. Most TFs related to upregulated genes mediate diverse functions such as negative regulation of antibody responses (CEBPA) [78], suppression of autoimmunity (TBX21, also termed T-bet) [79], and induction of FoxP3 expression (HIF1A) [80]. Additionally, by creating network analysis between all eight TFs in the CD4^+^ T cells (Supplementary Figure S4), we noted that the upregulated gene, *Fgf2*, linked with E2F4, and the downregulated *Cxcr2* linked with CEBPA were found relevant to autoimmunity [35]. While *Fgf2* is shown to be a critical cardioprotective factor [56], *Cxcr2* has been implicated in CNS autoimmunity [35]. Similarly, *Ifi204*, regulating the IRF7-mediated interferon production [81], and *Dapk1*, a proapoptotic gene [82,83], were linked with SMAD4. Likewise, ZBTB7a, including SMAD4, linked with the downregulated genes have been shown to contribute to self-tolerance or T cell anergy [84,85] and autoimmunity (MAZ and SP3) [86,87] (Supplementary Table S7). Of the eight TFs unique to CD8^+^ T cells, TCF4, RUNX1, NFE2L2, and BHLHE40, and ATF3, TCF3, SREBF1, and ID2, respectively, are associated with the upregulated and downregulated genes (Supplementary Table S7). Of these, TCF4 could restrict the development of Th1 and Th17 cells as noted in the EAE model [88], whereas BHLHE40 contributes to immune tolerance [89]. Finally, the network analysis created for all eight TFs in CD8^+^ T cells revealed two upregulated genes, *Cd5l* and *Itgax*, to be linked with SREBF1 and RUNX1, respectively (Supplementary Figure S4 and Supplementary Table S8). Of note, *Cd5l* is an apoptotic inhibitor of macrophage [90], whereas *Itgax*, encoding the integrin alpha X subunit of CD11c, has a role in cell adhesion and migration [91]. Taken together, the TF analysis revealed complex networks showing both similar and unique patterns between CD4^+^ and CD8^+^ T cells that have been implicated in Treg cell functions, T cell tolerance, and autoimmunity.

## 4. Conclusions

Using the purified CD4^+^ and CD8^+^ Tg T cells specific to Myhc-α 334–352, we performed bulk RNAseq and compared the transcriptome profiles between C57BL/6 and A/J mice. The analyses revealed several transcripts common to both subsets, including the genes specific to each subset. The DEG analysis revealed identical transcript profiles between CD4^+^ and CD8^+^ T cells related to immune functions such as cytokine production, apoptosis, cytotoxicity, and signaling. However, cytotoxicity genes, such as granzymes, were not expected in the CD4^+^ T cells. While the GO term enrichment analysis also revealed common pathways relevant to cytokines/chemokines and cytotoxicity, the downregulation of pathways related to autoimmune diseases, such as T1D and autoimmune thyroid disease, including GVHD allograft rejection and viral myocarditis, was noted in the C57BL/6 mice as determined by KEGG-enrichment analysis. Further, the GSEA of GO term analysis indicated a role for cytotoxicity genes such as *Klrs* in both CD4^+^ and CD8^+^ T cells. Although the TF analysis revealed a role for a number of TFs common to both subsets that regulate autoimmunity and Treg cell functions, we noted eight TFs to be unique in each subset. These TFs could mediate similar functions, such as maintenance of tolerance, suppression of autoimmunity, FoxP3 expression, and restricting the development of Th1 and Th17 cells [67,78,79,83,84,87,88]. Importantly, *Dapk1, Fgf2, Cxcr2*, and *Cd5l* were consistently noted across DEGs, enrichment, and TF subnetwork analyses. It is possible that alterations in the expression of genes promoting or suppressing T cell activation may occur simultaneously, but the skewed expression of a set of genes might influence the outcomes of T cell reactivity. Previously, single-cell RNA sequencing was performed to determine immune infiltrates in adjuvant-induced autoimmune and viral myocarditis models [66,92,93]. Our study focused on determining transcriptomic profiles of naïve cardiac myosin-specific T cells to understand variations in antigenic responses in the myocarditis-resistant (C57BL/6) and susceptible (A/J) Tg mice. While similar studies have not been reported to our knowledge, the availability of Tg mice expressing antigen-specific TCRs on CD4^+^ and CD8^+^ T cells in both mouse strains provided a useful framework to capture the differences described above. Although our data provide insights into the mechanisms of peripheral tolerance that may have translational significance, the determination of transcriptomic profiles before and after antigenic stimulation could provide additional leads on the potential molecular pathways critical to breaking tolerance. Likewise, it would be helpful to confirm the expression of genes at the protein level, which we could not verify due to the unavailability of Tg mice at the time of this writing. Finally, it is to be noted that we used the Tg T cells from generation four of the A/J Tg mice backcrossed with the C57BL/6 Tg mice containing the genetic composition of A/J mice of more than 90% [17]. It is possible that the differences noted in the current study may become more discernible in the fully backcrossed mice after ten generations. 

## Figures and Tables

**Table 1:** Enrichment Scores of GSEA-enriched GO and KEGG Analyses.

Pathways	NES	FDR
GSEA_GO analysis		
Positively enriched in CD4^+^ T cells		
Negative regulation by host of viral processes	2.273	0.04177
Transcription coactivator activity	2.121	0.05327
Negatively enriched in CD4^+^ T cells		
Negative regulation of heart contraction	−1.832	0.2275
Positive regulation of cardiac muscle hypertrophy	−1.882	0.1980
Positively enriched in CD8^+^ T cells		
Natural killer cell mediated cytotoxicity	1.979	0.08659
Negative regulation of extrinsic apoptotic signaling pathway in absence of ligand	1.893	0.12661
Positive regulation of I kappab kinase nf kappab signaling	1.927	0.09712
Positive regulation of interleukin 2 production	1.940	0.09835
Response to interferon beta	2.086	0.05416
GSEA_KEGG analysis		
Positively enriched in CD4^+^ T cells		
Natural killer cell mediated cytotoxicity	1.597	0.1258

## Data Availability

The original contributions presented in this study are included in the article/Supplementary Material. Further inquiries can be directed to the corresponding authors.

## Supplementary Materials

The following supporting information can be downloaded at https://www.mdpi.com/article/10.3390/biomedicines13112725/s1, Figure S1: The gating strategy used to sort CD4^+^ and CD8^+^ T cells from the Tg C57BL/6 (left) and A/J mice (right) by flow cytometry. Lymphocytes were obtained from a group of C57BL/6 and A/J Tg mice (*n* = 3 per group). CD3^+^ T cells were enriched from the lymphocytes using magnetic separation by negative selection and stained with anti-CD3^+^, anti-CD4^+^, anti-CD8^+^, anti-Vβ4, and Zombie violet. Viable singlet CD4^+^ (CD3^+^CD4^+^vβ4^+^Zombie^−^) and CD8 (CD3^+^CD4^+^vβ4^+^Zombie^−^) T cells were sorted by flow cytometry; Figure S2: Verification of the purity of the Tg CD4^+^ and CD8^+^ T cells after sorting by flow cytometry. The sorted CD4^+^ and CD8^+^ T cells were gated as CD3^+^CD4^+^vβ4+Zombie^−^ and CD3^+^CD4^+^vβ4+Zombie^−^ populations from singlets, respectively. The purity of each sorted population is shown on the left and right panels, respectively; Figure S3: Validation of selected DEGs by qPCR in CD4^+^ and CD8^+^ T cells from transgenic mice. The CD4^+^ and CD8^+^ T cells were sorted flow cytometrically from Tg mice. Total RNA was extracted, and qPCR was performed to assess the expression of selected DEGs in each T cell subset. Gene expression levels were normalized to Gapdh, and relative expressions were calculated using the 2^−(∆∆Ct)^ method. Data are presented as mean ± SEM, with *n* = 3 mice per group. Statistical significance was determined using a Student’s t-test; Figure S4: Transcription factor network analysis revealed TFs unique to both CD4^+^ and CD8^+^ T cells and TFs unique to each subset. (a) A subnetwork for eight selected TFs (E2F4, CEBPA, ZBTB7A, TBX21, SP3, SMAD4, POU2F2, and MAZ) and their common regulated genes in CD4^+^ T cells depicts a larger-scale subnetwork for differential gene expression between C57BL/6 and A/J mice. (b) A subnetwork for eight selected MRs (ATF3, BHLHE40, TCF3, TCF4, SREBF1, RUNX1, NFE2L2, ID2) and their common regulated genes in CD8^+^ T cells depicts a larger-scale subnetwork for differential gene expression between C57BL/6 and A/J mice; Table S1: Selected upregulated genes in the CD4^+^ T cell in C57BL/6 as compared to A/J mice; Table S2: Selected downregulated genes in the CD4^+^ T cell in C57BL/6 as compared to A/J mice; Table S3: Selected upregulated genes in the CD8^+^ T cell in C57BL/6 as compared to A/J mice; Table S4: Selected downregulated genes in the CD8^+^ T cell in C57BL/6 as compared to A/J mice; Table S5: The list of genes contributing to various processes revealed by GO enrichment analysis; Table S6: The list of genes contributing to various pathways revealed by KEGG enrichment analysis; Table S7: Selected the top 30 transcription factors linked to the transcriptomic profiles of CD4^+^ and CD8+ transgenic T cells of C57Bl/6 in relation to A/J mice; Table S8: Associations between transcription factors and the DEGs in the CD4^+^ and CD8^+^ T cell subsets.

## Abbreviations

The following abbreviations are used in this manuscript:

ATD	Autoimmune thyroid disease
Bmf	Bcl-2 modifying factor
Ct	Cycle threshold
Cxcr2	C-X-C Motif Chemokine Receptor 2
Cyp2r1	Cytochrome P450 Family 2 Subfamily R Member 1
DEG	Differentially expressed genes
EAE	Experimental autoimmune encephalomyelitis
EBV	Epstein–Barr virus
ES	Enrichment score
FDR	False discovery rate
FPKM	Fragments per kilobase of transcript per million mapped reads
Gapdh	Glyceraldehyde-3-phosphate dehydrogenase
GO	Gene Ontology
GRN	Gene regulatory networks
GSEA	Gene set enrichment analysis
GVHD	Graft-versus-host disease
GzmK	Granzyme K
HSV	Herpes simplex virus
IFN	Interferon
IL	Interleukin
Il12rb2	Interleukin 12 receptor subunit beta 2
KEGG	Kyoto Encyclopedia of Genes and Genomes
MHC	Major histocompatibility complex
Myhc	Myosin heavy chain
NES	Normalized enrichment score
Padj	Adjusted *P*-value
PCA	Principal component analysis
qPCR	Quantitative polymerase chain reaction
T1D	Type 1 diabetes
TCR	T cell receptor
TF	Transcription factor
Tg	Transgenic
Treg	T regulatory
