# Supplementary material for: Bulk RNAseq Analysis of Cardiac Myosin-Specific CD4+ and CD8+ T Cells Reveals Distinct Transcriptomic Profiles Between Myocarditis-Resistant and Susceptible Mice
Source: Biomedicines. 2025 Nov 6;13(11):2725. doi: 10.3390/biomedicines13112725 (PMC12650461; doi:10.3390/biomedicines13112725)
Supplement: Supplementary file 1 [file biomedicines-13-02725-s001.zip › Supplementary Figures.pdf]

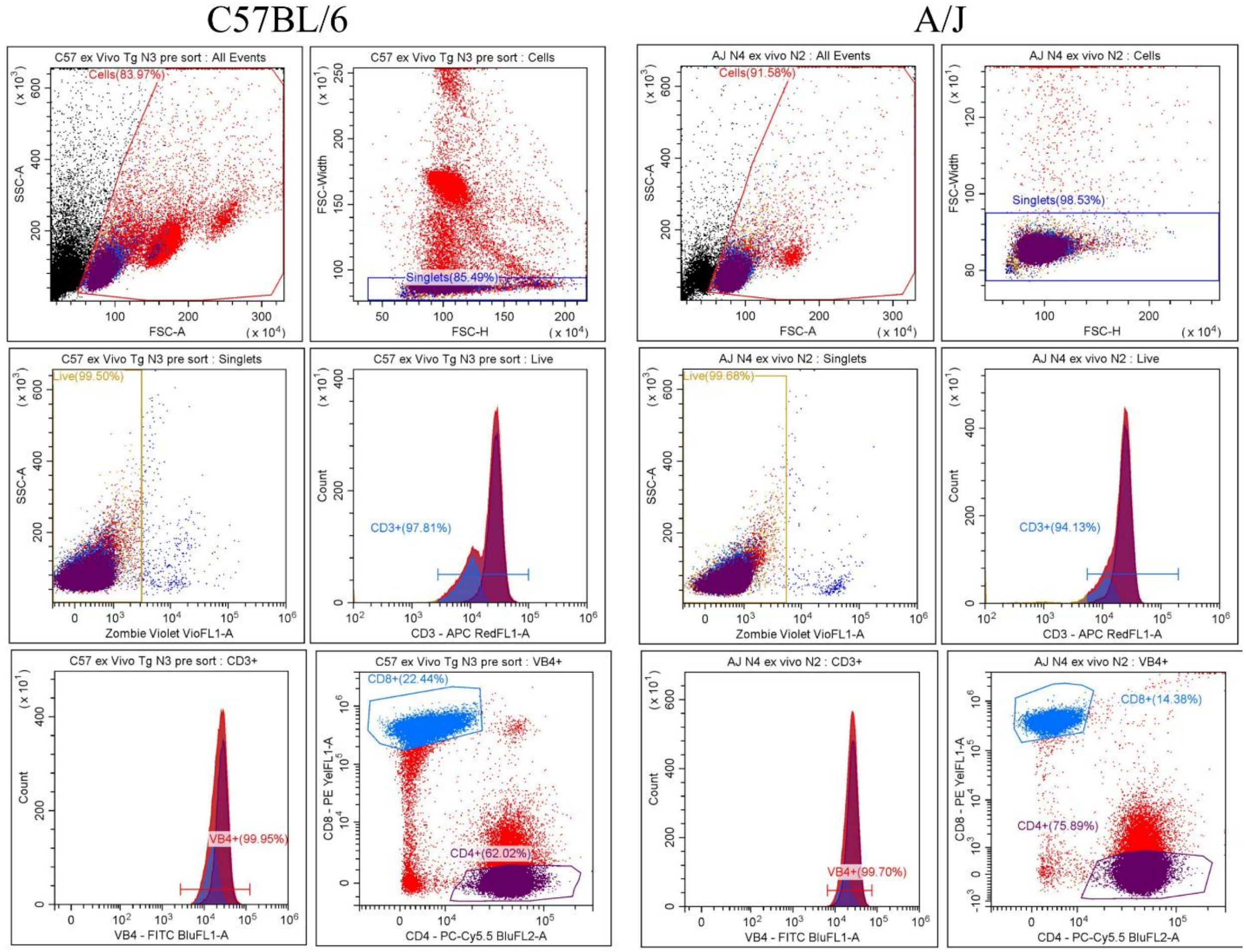

Supplementary Figure S1: The gating strategy used to sort CD4$^+$ and CD8$^+$ T cells from the Tg C57BL/6 (left) and A/J mice (right) by flow cytometry. Lymphocytes were obtained from a group of C57BL/6 and A/J Tg mice (*n* =3 per group). CD3$^+$ T cells were enriched from the lymphocytes using magnetic separation by negative selection and stained with anti-CD3$^+$, anti-CD4$^+$, anti-CD8$^+$, anti-Vβ4, and Zombie violet. Viable singlet CD4$^+$ (CD3$^+$CD4$^+$vβ4$^+$Zombie$^-$) and CD8 (CD3$^+$CD4$^+$vβ4$^+$Zombie$^-$) T cells were sorted by flow cytometry.

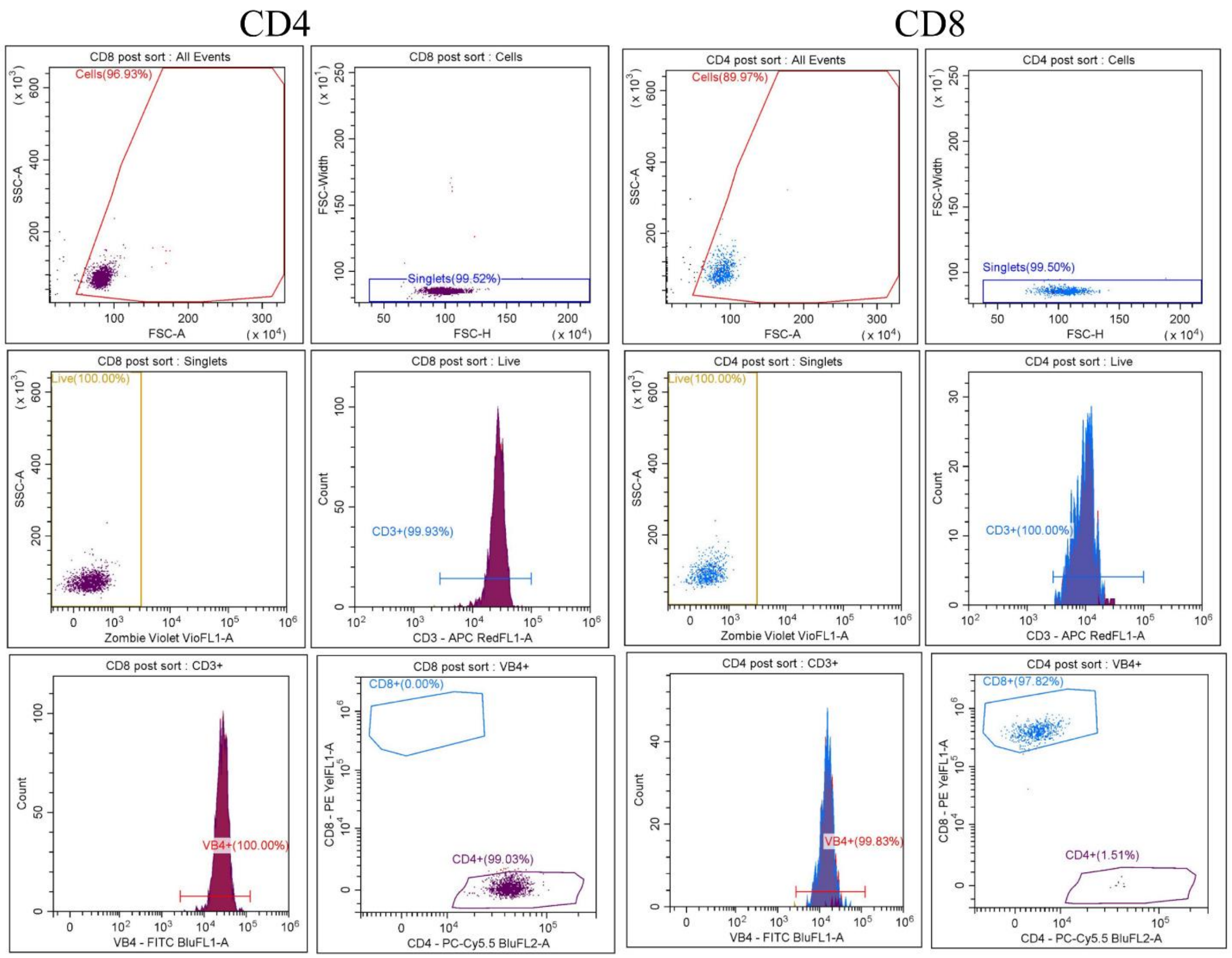

Supplementary Figure S2: Verification of the purity of the Tg CD4$^+$ and CD8$^+$ T cells after sorting by flow cytometry. The sorted CD4$^+$ and CD8$^+$ T cells were gated as CD3$^+$CD4$^+$vβ4$^+$Zombie$^-$ and CD3$^+$CD4$^+$vβ4$^+$Zombie$^-$ populations from singlets, respectively. The purity of each sorted population is shown on the left and right panels, respectively.

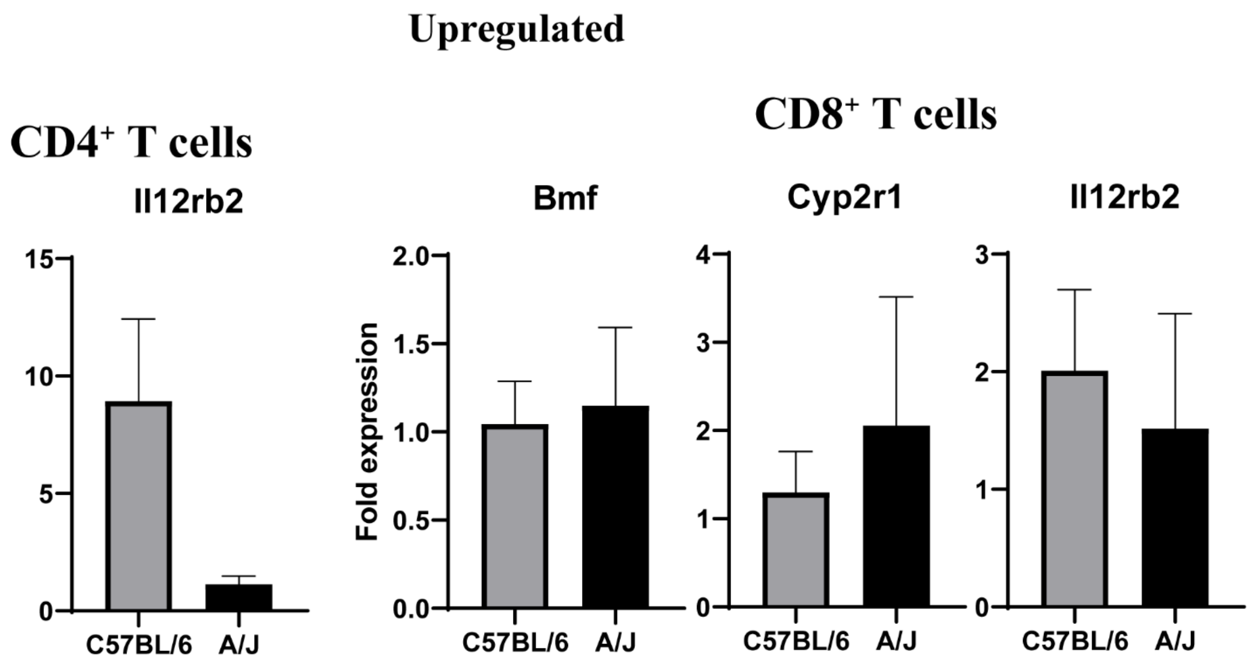

Supplementary Figure S3: Validation of selected DEGs by qPCR in CD4$^+$ and CD8$^+$ T cells from transgenic mice. The CD4$^+$ and CD8$^+$ T cells were sorted flow cytometrically from Tg mice. Total RNA was extracted, and qPCR was performed to assess the expression of selected DEGs in each T cell subset. Gene expression levels were normalized to *Gapdh*, and relative expressions were calculated using the $2^{-(\Delta\Delta Ct)}$ method. Data are presented as mean ± SEM, with n = 3 mice per group. Statistical significance was determined using a Student's t-test.

a) CD4^{+} T cells

b) CD8^{+} T cells

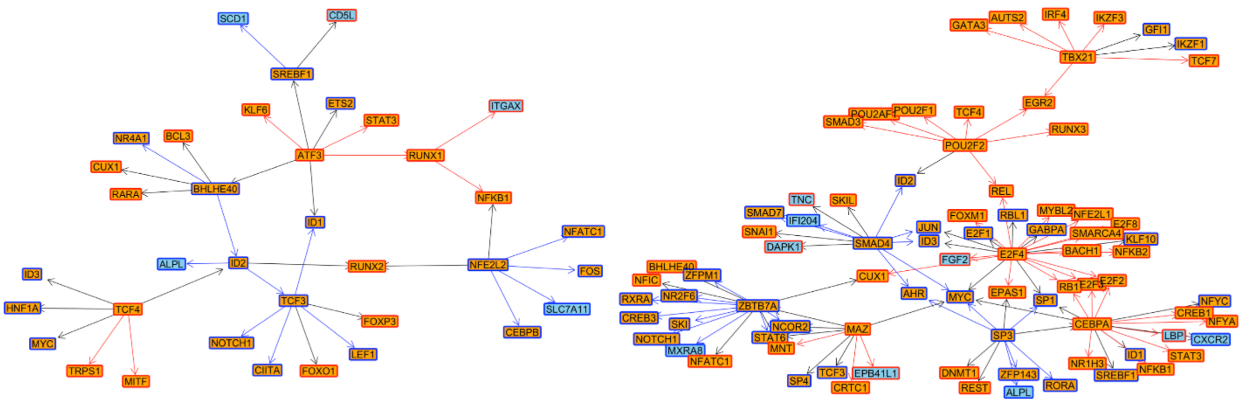

Supplementary Figure S4: Transcription factor network analysis revealed TFs unique to both CD4^{+} and CD8^{+} T cells and TFs unique to each subset. (**a**) A subnetwork for eight selected TFs (E2F4, CEBPA, ZBTB7A, TBX21, SP3, SMAD4, POU2F2, and MAZ) and their common regulated genes in CD4^{+} T cells depicts a larger-scale subnetwork for differential gene expression between C57BL/6 and A/J mice. (**b**) A subnetwork for eight selected MRs (ATF3, BHLHE40, TCF3, TCF4, SREBF1, RUNX1, NFE2L2, ID2) and their common regulated genes in CD8^{+} T cells depicts a larger-scale subnetwork for differential gene expression between C57BL/6 and A/J mice.
