# Supplementary material for: Bulk RNAseq Analysis of Cardiac Myosin-Specific CD4+ and CD8+ T Cells Reveals Distinct Transcriptomic Profiles Between Myocarditis-Resistant and Susceptible Mice
Source: Biomedicines. 2025 Nov 6;13(11):2725. doi: 10.3390/biomedicines13112725 (PMC12650461; doi:10.3390/biomedicines13112725)
Supplement: Supplementary file 1 [file biomedicines-13-02725-s001.zip › Supplementary Tables.pdf]

Supplementary Table S1. Selected upregulated genes in the $CD4^+$ T cells in C57BL/6 as compared to A/J mice

Log2Fold	Padj	Gene Name	Gene Description
10.5977	1.03E-16	Lad1	ladinin [Source:MGI Symbol;Acc:MGI:109343]
10.15318	9.49E-21	DAPK1	death associated protein kinase 1 [Source:MGI Symbol;Acc:MGI:1916885]
6.976735	3.77E-88	Trim30d	tripartite motif-containing 30D [Source:MGI Symbol;Acc:MGI:3035181]
8.60640820	2.37e-09	Cxcr1	chemokine (C-X-C motif) receptor 1 [Source:MGI Symbol;Acc:MGI:2448715]
6.870158	5.51E-06	Ccl27a	chemokine (C-C motif) ligand 27A [Source:MGI Symbol;Acc:MGI:1343459]
8.312776	2.12e-08	Tnnt2	troponin T2, cardiac [Source:MGI Symbol;Acc:MGI:104597]
6.741448	4.58E-89	Acsf2	acyl-CoA synthetase family member 2 [Source:MGI Symbol;Acc:MGI:2388287]
6.427191	0.000389	Vcan	versican [Source:MGI Symbol;Acc:MGI:102889]
6.125942	0.001703	Cdh11	cadherin 11 [Source:MGI Symbol;Acc:MGI:99217]
6.088598	0.002183	Trim30b	tripartite motif-containing 30B [Source:MGI Symbol;Acc:MGI:4821256]
6.074335	0.002761	Sox5	SRY (sex determining region Y)-box 5 [Source:MGI Symbol;Acc:MGI:98367]
5.922226	0.001078	Dnase1l3	deoxyribonuclease 1-like 3 [Source:MGI Symbol;Acc:MGI:1314633]
5.706566	0.009345	Khdc1a	KH domain containing 1A [Source:MGI Symbol;Acc:MGI:2676610]
5.50985	0.024595	Sash1	SAM and SH3 domain containing 1 [Source:MGI Symbol;Acc:MGI:1917347]
4.979219	0.002664	Ly6m	lymphocyte antigen 6 complex, locus M [Source:MGI Symbol;Acc:MGI:1914288]
4.734940	1.93082e-06	Gzmk	granzyme K [Source:MGI Symbol;Acc:MGI:1298232]
4.69957	1.93E-174	Rpl21	ribosomal protein L21 [Source:MGI Symbol;Acc:MGI:1278340]
4.689993	0.006739	Hopxos	HOP homeobox, opposite strand [Source:MGI Symbol;Acc:MGI:3801917]
4.539278	0.020688	Syk	spleen tyrosine kinase [Source:MGI Symbol;Acc:MGI:99515]
4.378493	0	Ifi213	interferon activated gene 213 [Source:MGI Symbol;Acc:MGI:3695276]
4.096276	0.047464	Ifi211	interferon activated gene 211 [Source:MGI Symbol;Acc:MGI:3041120]
4.039355	5.65E-13	G0s2	G0/G1 switch gene 2 [Source:MGI Symbol;Acc:MGI:1316737]
3.841729	9.99E-06	Map3k19	mitogen-activated protein kinase kinase kinase 19 [Source:MGI Symbol;Acc:MGI:1203481]
3.810392	6.49E-18	Bmf	BCL2 modifying factor [Source:MGI Symbol;Acc:MGI:2176433]
3.755769	1.13E-10	Cyp2r1	cytochrome P450, family 2, subfamily r, polypeptide 1 [Source:MGI Symbol;Acc:MGI:2449771]
3.73023	0.008753	Zg16	zymogen granule protein 16 [Source:MGI Symbol;Acc:MGI:1916286]
3.661381	1.66E-09	Hif3a	hypoxia inducible factor 3, alpha subunit [Source:MGI Symbol;Acc:MGI:1859778]
3.61048	0.048018	Ltbp2	latent transforming growth factor beta binding protein 2 [Source:MGI Symbol;Acc:MGI:99502]
3.446422	0.002846	Il17rd	interleukin 17 receptor D [Source:MGI Symbol;Acc:MGI:2159727]

3.276488	0.005332	Scart1	scavenger receptor family member expressed on T cells 1 [Source:MGI Symbol;Acc:MGI:2443796]
3.23532	4.83E-67	Arhgap29	Rho GTPase activating protein 29 [Source:MGI Symbol;Acc:MGI:2443818]
3.219264	3.07E-279	Pdk1	pyruvate dehydrogenase kinase, isoenzyme 1 [Source:MGI Symbol;Acc:MGI:1926119]
3.045398	1.40E-08	Klrk1	killer cell lectin-like receptor subfamily K, member 1 [Source:MGI Symbol;Acc:MGI:1196250]
2.872668	2.51E-127	Ifi206	interferon activated gene 206 [Source:MGI Symbol;Acc:MGI:3646410]
2.439724	1.81E-06	Cxcr1	chemokine (C-X-C motif) receptor 1 [Source:MGI Symbol;Acc:MGI:2448715]
2.415366	3.49E-20	Fgf2	fibroblast growth factor 2 [Source:MGI Symbol;Acc:MGI:95516]
2.414945	0.036863	Ly6e	lymphocyte antigen 6 complex, locus E [Source:MGI Symbol;Acc:MGI:106651]
2.375595	4.22E-05	Aqp9	aquaporin 9 [Source:MGI Symbol;Acc:MGI:1891066]
2.335751	0.011942	Klf4	Kruppel-like factor 4 (gut) [Source:MGI Symbol;Acc:MGI:1342287]
2.25484	1.57E-07	Nfam1	Nfat activating molecule with ITAM motif 1 [Source:MGI Symbol;Acc:MGI:1921289]
2.100201	5.56E-35	Gbp6	guanylate binding protein 6 [Source:MGI Symbol;Acc:MGI:2140937]
2.057497	0.031889	Deptor	DEP domain containing MTOR-interacting protein [Source:MGI Symbol;Acc:MGI:2146322]
2.055696	7.63E-07	Hspa12a	heat shock protein 12A [Source:MGI Symbol;Acc:MGI:1920692]
2.020573	1.25E-05	Itga10	integrin, alpha 10 [Source:MGI Symbol;Acc:MGI:2153482]

Supplementary Table S2. Selected downregulated genes in the CD4$^+$ T cells in C57BL/6 as compared to A/J mice

log2Fold	Padj	Gene Name	Gene Description
-10.1551	4.56E-15	Arntl2	aryl hydrocarbon receptor nuclear translocator-like 2 [Source:MGI Symbol;Acc:MGI:2684845]
-8.08094	1.97E-08	Cxcr2	chemokine (C-X-C motif) receptor 2 [Source:MGI Symbol;Acc:MGI:105303]
-8.00145	1.81E-08	Ifi44l	interferon-induced protein 44 like [Source:MGI Symbol;Acc:MGI:95975]
-6.88375	4.33E-105	Gbp2b	guanylate binding protein 2b [Source:MGI Symbol;Acc:MGI:95666]
-6.73122	4.92E-129	Pde5a	phosphodiesterase 5A, cGMP-specific [Source:MGI Symbol;Acc:MGI:2651499]
-6.15015	0.0034252	Ccl1	chemokine (C-C motif) ligand 1 [Source:MGI Symbol;Acc:MGI:98258]
-4.61391	0.049897	Ripk4	receptor-interacting serine-threonine kinase 4 [Source:MGI Symbol;Acc:MGI:1919638]
-3.86151	0.009553	Scd1	stearoyl-Coenzyme A desaturase 1 [Source:MGI Symbol;Acc:MGI:98239]
-3.82595	4.11E-21	Trim16	tripartite motif-containing 16 [Source:MGI Symbol;Acc:MGI:2137356]
-3.80096	3.67E-32	Eno1b	enolase 1B, retrotransposed [Source:MGI Symbol;Acc:MGI:3648653]
-3.54272	0.004012	Cd244a	CD244 molecule A [Source:MGI Symbol;Acc:MGI:109294]
-3.38854	0.001963	Ccn4	cellular communication network factor 4 [Source:MGI Symbol;Acc:MGI:1197008]
-3.00424	0.005806	Fgf7	fibroblast growth factor 7 [Source:MGI Symbol;Acc:MGI:95521]
-2.84131	0.000171	Tlr4	toll-like receptor 4 [Source:MGI Symbol;Acc:MGI:96824]
-2.72518	6.80E-07	Wnt3	wingless-type MMTV integration site family, member 3 [Source:MGI Symbol;Acc:MGI:98955]
-2.48341	3.64E-06	Stra6	stimulated by retinoic acid gene 6 [Source:MGI Symbol;Acc:MGI:107742]
-2.29315	2.59E-21	Rab4a	RAB4A, member RAS oncogene family [Source:MGI Symbol;Acc:MGI:105069]
-2.28833	0.001984	Sapcd1	suppressor APC domain containing 1 [Source:MGI Symbol;Acc:MGI:2388100]
-2.24784	0.042099	Plce1	phospholipase C, epsilon 1 [Source:MGI Symbol;Acc:MGI:1921305]
-2.21594	0.03283	Rab26os	RAB26, member RAS oncogene family, opposite strand [Source:MGI Symbol;Acc:MGI:1922864]
-2.18555	7.74E-11	Tnfrsf19	tumor necrosis factor receptor superfamily, member 19 [Source:MGI Symbol;Acc:MGI:1352474]
-2.02558	4.99E-11	Fbxo27	F-box protein 27 [Source:MGI Symbol;Acc:MGI:2685007]
-1.97513	6.89E-05	Gzmb	granzyme B [Source:MGI Symbol;Acc:MGI:109267]

Supplementary Table S3. Selected upregulated genes in the CD8$^+$ T cells in C57BL/6 as compared to A/J mice

log2Fold	Padj	Gene Name	Gene Description
8.6161416	5.10132631718498e-10	Lbp	lipopolysaccharide binding protein [Source:MGI Symbol;Acc:MGI:1098776]
8.4906901	6.0037959152385e-09	Chaer1	cardiac hypertrophy associated epigenetic regulator 1 [Source:MGI Symbol;Acc:MGI:5624990]
7.6984743	1.00181869430859e-15	Klra10	killer cell lectin-like receptor subfamily A, member 10 [Source:MGI Symbol;Acc:MGI:1321093]
7.593171	5.02106516759568e-07	Ccl27a	chemokine (C-C motif) ligand 27A [Source:MGI Symbol;Acc:MGI:1343459]
7.534940	0.000145247	Cd5l	CD5 antigen-like [Source:MGI Symbol;Acc:MGI:1334419]
7.3254845	2.91816519849434e-06	Chaer1	cardiac hypertrophy associated epigenetic regulator 1 [Source:MGI Symbol;Acc:MGI:5624990]
7.1836179	1.42043089547379e-06	Klra6	killer cell lectin-like receptor, subfamily A, member 6 [Source:MGI Symbol;Acc:MGI:101902]
7.0477935	4.97527839135986e-05	Gzmk	granzyme K [Source:MGI Symbol;Acc:MGI:1298232]
6.987837	3.54840699740698e-05	Strc	stereocilin [Source:MGI Symbol;Acc:MGI:2153816]
6.8785733	0	Mndal	myeloid nuclear differentiation antigen like [Source:MGI Symbol;Acc:MGI:3780953]
6.7809198	6.25593999964612e-77	Prss12	protease, serine 12 neurotrypsin (motopsin) [Source:MGI Symbol;Acc:MGI:1100881]
6.7185355	2.24334576915235e-14	Klra1	killer cell lectin-like receptor, subfamily A, member 1 [Source:MGI Symbol;Acc:MGI:101907]
6.1035287	0.022483709	Foxq1	forkhead box Q1 [Source:MGI Symbol;Acc:MGI:1298228]
6.0949119	1.34277351200083e-08	Klra7	killer cell lectin-like receptor, subfamily A, member 7 [Source:MGI Symbol;Acc:MGI:101901]
5.9763043	0.004414	Bcl2l14	BCL2-like 14 (apoptosis facilitator) [Source:MGI Symbol;Acc:MGI:1914063]
5.893774	0.000676	Klri2	killer cell lectin-like receptor family I member 2 [Source:MGI Symbol;Acc:MGI:2443965]
5.7565078	6.32164250298552e-49	Rasgrf2	RAS protein-specific guanine nucleotide-releasing factor 2 [Source:MGI Symbol;Acc:MGI:109137]
5.7419235	0.013531	Muc3a	mucin 3A, cell surface associated [Source:MGI Symbol;Acc:MGI:3588263]
5.6604491	2.23728058150238e-256	Gvin3	GTPase, very large interferon inducible, family member 3 [Source:MGI Symbol;Acc:MGI:3584360]
5.5905252	0.024415	Cacna1i	calcium channel, voltage-dependent, alpha 1I subunit [Source:MGI Symbol;Acc:MGI:2178051]
5.4855586	0.020819	Ly6d	lymphocyte antigen 6 complex, locus D [Source:MGI Symbol;Acc:MGI:96881]
5.3068958	0.002125	Klra9	killer cell lectin-like receptor subfamily A, member 9 [Source:MGI Symbol;Acc:MGI:1321153]
4.9379443	0.000466	Klra8	killer cell lectin-like receptor, subfamily A, member 8 [Source:MGI Symbol;Acc:MGI:102968]
4.9254923	5.0415787851982e-12	Cd93	CD93 antigen [Source:MGI Symbol;Acc:MGI:106664]
4.6950198	0.000216885	Fcgr3	Fc receptor, IgG, low affinity III [Source:MGI Symbol;Acc:MGI:95500]

4.6711589	0.000739	Itga1	integrin alpha 1 [Source:MGI Symbol;Acc:MGI:96599]
4.4729998	9.50433140346277e-257	Ifi213	interferon activated gene 213 [Source:MGI Symbol;Acc:MGI:3695276]
4.216528	5.14283460548106e-09	Klre1	killer cell lectin-like receptor family E member 1 [Source:MGI Symbol;Acc:MGI:2662547]
4.1670683	0.007432	Tifa	TRAF-interacting protein with forkhead-associated domain [Source:MGI Symbol;Acc:MGI:2182965]
3.8613092	4.11325307943803e-10	Nrp1	neuropilin 1 [Source:MGI Symbol;Acc:MGI:106206]
3.4171101	0.006567459	Cyp17a1	cytochrome P450, family 17, subfamily a, polypeptide 1 [Source:MGI Symbol;Acc:MGI:88586]
3.4062127	4.6540575584961e-19	Bmf	BCL2 modifying factor [Source:MGI Symbol;Acc:MGI:2176433]
3.3468047	3.6637179605596e-19	Klra3	killer cell lectin-like receptor, subfamily A, member 3 [Source:MGI Symbol;Acc:MGI:101905]
3.2415986	0.002631	Itga5	integrin alpha 5 (fibronectin receptor alpha) [Source:MGI Symbol;Acc:MGI:96604]
3.0937996	0.0403439	Fap	fibroblast activation protein [Source:MGI Symbol;Acc:MGI:109608]
2.9569342	1.6957983328365e-59	Ifi214	interferon activated gene 214 [Source:MGI Symbol;Acc:MGI:3584522]
3.074409	0.011209906	Itga5	integrin alpha 5 (fibronectin receptor alpha) [Source:MGI Symbol;Acc:MGI:96604]
2.8142259	0.000539	Cyp2r1	cytochrome P450, family 2, subfamily r, polypeptide 1 [Source:MGI Symbol;Acc:MGI:2449771]
2.8703277	2.80975430e-42	Itgb3	integrin beta 3 [Source:MGI Symbol;Acc:MGI:96612]
2.5108454	0.000101	Ctla2b	cytotoxic T lymphocyte-associated protein 2 beta [Source:MGI Symbol;Acc:MGI:88555]
2.2817964	0.000947	Ly6e	lymphocyte antigen 6 complex, locus E [Source:MGI Symbol;Acc:MGI:106651]
2.2694529	3.12800522504418e-15	Fgf2	fibroblast growth factor 2 [Source:MGI Symbol;Acc:MGI:95516]
2.1899349	4.13327353128686e-30	Gbp6	guanylate binding protein 6 [Source:MGI Symbol;Acc:MGI:2140937]
2.1547209	6.24523412984202e-13	Tlr7	toll-like receptor 7 [Source:MGI Symbol;Acc:MGI:2176882]
2.1473322	1.74393504306207e-06	Lilrb4b	leukocyte immunoglobulin-like receptor, subfamily B, member 4B [Source:MGI Symbol;Acc:MGI:102702]
2.1227677	0.026123	Myadm	myeloid-associated differentiation marker [Source:MGI Symbol;Acc:MGI:1355332]
2.1133693	9.58437907508189e-06	Bcl2l2	BCL2-like 2 [Source:MGI Symbol;Acc:MGI:108052]
2.1081493	9.81415132578918e-30	Sipa1l2	signal-induced proliferation-associated 1 like 2 [Source:MGI Symbol;Acc:MGI:2676970]
2.054648	0.000287	Il12rb2	interleukin 12 receptor, beta 2 [Source:MGI Symbol;Acc:MGI:1270861]
2.0150785	0.000678	Lilrb4a	leukocyte immunoglobulin-like receptor, subfamily B, member 4A [Source:MGI Symbol;Acc:MGI:102701]

Supplementary Table S4. Selected downregulated genes in the CD8$^+$ T cells in C57BL/6 as compared to A/J mice

Log2Fold	Padj	Gene Name	Gene Description
-12.013	5.8047907975e-22	Gbp2b	guanylate binding protein 2b [Source:MGI Symbol;Acc:MGI:95666]
-9.82433	6.83603243409e-14	Ccnb1ip1	cyclin B1 interacting protein 1 [Source:MGI Symbol;Acc:MGI:2685134]
-9.31423	1.09538067431e-12	Mtus1	mitochondrial tumor suppressor 1 [Source:MGI Symbol;Acc:MGI:2142572]
-7.85914	2.70805668271e-07	Kcnf1	potassium voltage-gated channel, subfamily F, member 1 [Source:MGI Symbol;Acc:MGI:2687399]
-7.13333	0.001408107	Arntl2	aryl hydrocarbon receptor nuclear translocator-like 2 [Source:MGI Symbol;Acc:MGI:2684845]
-6.55365	4.98295190739e-16	H2-T3	histocompatibility 2, T region locus 3 [Source:MGI Symbol;Acc:MGI:95959]
-5.65548	4.50938773140e-38	Ltk	leukocyte tyrosine kinase [Source:MGI Symbol;Acc:MGI:96840]
-5.63848	8.64617860313e-05	Sp6	trans-acting transcription factor 6 [Source:MGI Symbol;Acc:MGI:1932575]
-5.61653	9.54743717934e-16	Ly6c1	lymphocyte antigen 6 complex, locus C1 [Source:MGI Symbol;Acc:MGI:96882]
-5.43885	0.001755918	Cxcr2	chemokine (C-X-C motif) receptor 2 [Source:MGI Symbol;Acc:MGI:105303]
-5.36917	3.30560553496e-21	Ifi204	interferon activated gene 204 [Source:MGI Symbol;Acc:MGI:96429]
-5.26584	0.009166	Cxcr2	chemokine (C-X-C motif) receptor 2 [Source:MGI Symbol;Acc:MGI:105303]
-5.19225	0.015165	Nlrp3	NLR family, pyrin domain containing 3 [Source:MGI Symbol;Acc:MGI:2653833]
-4.41787	9.439196857377e-07	Capn9	calpain 9 [Source:MGI Symbol;Acc:MGI:1920897]
-4.21106	4.988637831119e-08	Tlr4	toll-like receptor 4 [Source:MGI Symbol;Acc:MGI:96824]
-4.19265	0.000885	H2-Q1	histocompatibility 2, Q region locus 1 [Source:MGI Symbol;Acc:MGI:95928]
-3.97492	0.005476	Tal1	T cell acute lymphocytic leukemia 1 [Source:MGI Symbol;Acc:MGI:98480]
-3.61360	0.0157323	Cox6a2	cytochrome c oxidase subunit 6A2 [Source:MGI Symbol;Acc:MGI:104649]
-3.16435	0.02022	Fgf7	fibroblast growth factor 7 [Source:MGI Symbol;Acc:MGI:95521]
-2.83694	7.064286227804e-34	Stc2	stanniocalcin 2 [Source:MGI Symbol;Acc:MGI:1316731]
-2.79694	3.69302776617e-37	P2rx7	purinergic receptor P2X, ligand-gated ion channel, 7 [Source:MGI Symbol;Acc:MGI:1339957]
-2.51212	0.004811	Zfp30	zinc finger protein 30 [Source:MGI Symbol;Acc:MGI:99178]

-2.42566	3.59968514572592e-12	Fbxo27	F-box protein 27 [Source:MGI Symbol;Acc:MGI:2685007]
-2.36462	0.000229	Ikbip	IKBKB interacting protein [Source:MGI Symbol;Acc:MGI:1914704]
-2.35013	0.048075	H4c8	H4 clustered histone 8 [Source:MGI Symbol;Acc:MGI:2448427]
-2.32042	4.098015129e-21	Hmga1b	high mobility group AT-hook 1B [Source:MGI Symbol;Acc:MGI:96161]
-2.32016	5.609231461072e-45	Fbxo17	F-box protein 17 [Source:MGI Symbol;Acc:MGI:1354707]
-2.21412	0.000106	Itga7	integrin alpha 7 [Source:MGI Symbol;Acc:MGI:102700]
-2.0748	0.041461	Mapk8ip1	mitogen-activated protein kinase 8 interacting protein 1 [Source:MGI Symbol;Acc:MGI:1309464]

Supplementary Table S5. The list of genes contributing to various processes revealed by GO enrichment analysis

GO ID	Description	Gene Ratio	P value	Padj	Gene ID	Gene Name	Count
**a) Upregulated pathways within CD4⁺ T cells in C57BL/6 as compared to A/J**							
GO:0048525	Negative regulation of viral process	13/487	5.39e-06	0.020491014	ENSMUSG00000066258/ ENSMUSG00000060441/ ENSMUSG00000057596/ ENSMUSG00000020641/ ENSMUSG00000052776/ ENSMUSG00000041827/ ENSMUSG00000006369/ ENSMUSG00000024610/ ENSMUSG00000009585/ ENSMUSG00000052749/ ENSMUSG00000017002/ ENSMUSG00000032690/ ENSMUSG00000022587	Trim12a/Trim5/Trim30d/Rsad2/Oas1a/Oasl1/Fbln1/Cd74/ Apobec3/Trim30b/Slpi/ Oas2/Ly6e	13
GO:0002218	Activation of innate immune response	22/487	1.642e-05	0.023643681	ENSMUSG00000090272/ ENSMUSG00000066258/ ENSMUSG00000073491/ ENSMUSG00000060441/ ENSMUSG00000057596/ ENSMUSG00000021678/ ENSMUSG00000037849/ ENSMUSG00000039997/ ENSMUSG00000022899/ ENSMUSG00000070501/ ENSMUSG00000030165/ ENSMUSG00000016024/ ENSMUSG00000020641/ ENSMUSG00000030149/ ENSMUSG00000052776/ ENSMUSG00000041827/ ENSMUSG00000074682/ ENSMUSG00000027995/ ENSMUSG00000026536/ ENSMUSG00000047798/ ENSMUSG00000052749/ ENSMUSG00000021457	Mndal/Trim12a/Ifi213/Trim5/Trim30d/F2rl1/Ifi206/Ifi203/Slc15a2/ Ifi214/Klrd1/Lbp/Rsad2/Klrk1/Oas1a/Oasl1/Zcchc3/Tlr2/ Ifi211/Cd300lf/Trim30b/Syk	22
GO:0032602	Chemokine production	13/487	1.869e-05	0.023643681	ENSMUSG00000021678/ ENSMUSG00000016024/ ENSMUSG00000052776/ ENSMUSG00000029135/ ENSMUSG00000015452/ ENSMUSG00000024610/ ENSMUSG00000112023/ ENSMUSG00000027995/ ENSMUSG00000112148/ ENSMUSG00000014158/ ENSMUSG00000003032/ ENSMUSG00000021457/ ENSMUSG00000040249	F2rl1/Lbp/ Oas1a/Fosl2/Ager/Cd74/Lilrb4b/Tlr2/Lilrb4a/ Trpv4/Klf4/ Syk/Lrp1	13

GO:0032642	Regulation of chemokine production	12/487	4.79e-05	0.036123949	ENSMUSG00000021678/ ENSMUSG00000016024/ ENSMUSG00000052776/ ENSMUSG00000015452/ ENSMUSG00000024610/ ENSMUSG00000112023/ ENSMUSG00000027995/ ENSMUSG00000112148/ ENSMUSG00000014158/ ENSMUSG00000003032/ ENSMUSG00000021457/ ENSMUSG00000040249	F2rl1/Lbp/Oas1a/Ager/Cd74/Lilrb4b/Tlr2/Lilrb4a/Trpv4/Klf4/Syk/Lrp1	12
GO:0035458	cellular response to interferon-beta	9/487	5.71e-05	0.036123949	ENSMUSG00000090272/ ENSMUSG00000073491/ ENSMUSG00000037849/ ENSMUSG00000039997/ ENSMUSG00000070501/ ENSMUSG00000052776/ ENSMUSG00000104713/ ENSMUSG00000024617/ ENSMUSG00000026536	Mndal/Ifi213/Ifi206/Ifi203/Ifi214/Oas1a/Gbp6/Camk2a/Ifi211	9
**b)**	**Downregulated pathways within CD4^{+} T cells in C57BL/6 as compared to A/J**						
GO:0002484	antigen processing and presentation of endogenous peptide antigen via MHC class I via ER pathway	6/256	4.819e-06	0.001262192	ENSMUSG00000079507/ ENSMUSG00000054128/ ENSMUSG00000079492/ ENSMUSG00000073402/ ENSMUSG00000075297/ ENSMUSG00000073406	H2-Q1/H2-T3/Gm11127/Gm8909/H60b/H2-Bl	6
GO:0001913	T cell mediated cytotoxicity	9/256	7.498e-07	0.000442164	ENSMUSG00000079507/ ENSMUSG00000054128/ ENSMUSG00000079492/ ENSMUSG00000073402/ ENSMUSG00000041750/ ENSMUSG00000015437/ ENSMUSG00000075297/ ENSMUSG00000073406/ ENSMUSG00000036322	H2-Q1/H2-T3/Gm11127/Gm8909/Cd1d2/Gzmb/H60b/H2-Bl/H2-Ea	9
GO:0001906	cell killing	15/256	7.437e-07	0.000442164	ENSMUSG00000079507/ ENSMUSG00000040264/ ENSMUSG00000054128/ ENSMUSG00000079492/ ENSMUSG00000028268/ ENSMUSG00000073402/ ENSMUSG00000032021/ ENSMUSG00000041750/ ENSMUSG00000015437/ ENSMUSG00000050335/ ENSMUSG00000075297/ ENSMUSG00000030154/ ENSMUSG00000073406/ ENSMUSG00000020702/ ENSMUSG00000036322	H2-Q1/Gbp2b/H2-T3/Gm11127/Gbp3/Gm8909/Crtam/Cd1d2/Gzmb/Lgals3/H60b/Klrb1f/H2-Bl/Ccl1/H2-Ea	15
GO:0002483	antigen processing and presentation of endogenous peptide antigen	7/256	1.012e-06	0.000442164	ENSMUSG00000079507/ ENSMUSG00000054128/ ENSMUSG00000079492/ ENSMUSG00000073402/ ENSMUSG00000075297/ ENSMUSG00000073406/ ENSMUSG00000036322	H2-Q1/H2-T3/Gm11127/Gm8909/H60b/H2-Bl/H2-Ea	7

GO:0001909	leukocyte-mediated cytotoxicity	11/256	1.55762313105151e-05	0.002549634	ENSMUSG00000079507/ENSMUSG00000054128/ENSMUSG00000079492/ENSMUSG00000073402/ENSMUSG00000032021/ENSMUSG00000041750/ENSMUSG00000015437/ENSMUSG00000075297/ENSMUSG00000030154/ENSMUSG00000073406/ENSMUSG00000036322	H2-Q1/H2-T3/Gm11127/Gm8909/Crtam/Cd1d2/Gzmb/H60b/Klrb1f/H2-Bl/H2-Ea	11
GO:0042267	natural killer cell mediated cytotoxicity	7/256	0.000494074	0.047925146	ENSMUSG00000079507/ENSMUSG00000054128/ENSMUSG00000032021/ENSMUSG00000015437/ENSMUSG00000075297/ENSMUSG00000030154/ENSMUSG00000073406	H2-Q1/H2-T3/Crtam/Gzmb/H60b/Klrb1f/H2-Bl	7
GO:2001185	regulation of CD8-positive, alpha-beta T cell activation	5/256	0.000270904	0.028379879	ENSMUSG00000079507/ENSMUSG00000054128/ENSMUSG00000032021/ENSMUSG00000004709/ENSMUSG00000073406	H2-Q1/H2-T3/Crtam/Cd244a/H2-Bl	5
GO:0019882	antigen processing and presentation	9/256	6.57183498144821e-05	0.008196017	ENSMUSG00000079507/ENSMUSG00000054128/ENSMUSG00000019478/ENSMUSG00000079492/ENSMUSG00000073402/ENSMUSG00000041750/ENSMUSG00000075297/ENSMUSG00000073406/ENSMUSG00000036322	H2-Q1/H2-T3/Rab4a/Gm11127/Gm8909/Cd1d2/H60b/H2-Bl/H2-Ea	9
GO:0042605	peptide antigen binding	11/256	1.80878962915046e-09	8.10337753859406e-07	ENSMUSG00000094619/ENSMUSG00000079507/ENSMUSG00000054128/ENSMUSG00000079492/ENSMUSG00000094023/ENSMUSG00000073402/ENSMUSG00000094766/ENSMUSG00000093801/ENSMUSG00000073406/ENSMUSG00000036322/ENSMUSG00000105629	Trav14d-3-dv8/H2-Q1/H2-T3/Gm11127/Trav7d-4/Gm8909/Trav7-4/Trav14n-2/H2-Bl/H2-Ea/Trav7n-5	11
GO:0003823	antigen binding	12/256	1.68610808415325e-07	3.77688210850328e-05	ENSMUSG00000094619/ENSMUSG00000079507/ENSMUSG00000054128/ENSMUSG00000079492/ENSMUSG00000094023/ENSMUSG00000073402/ENSMUSG00000094766/ENSMUSG00000041750/ENSMUSG00000093801/ENSMUSG00000073406/ENSMUSG00000036322/ENSMUSG00000105629	Trav14d-3-dv8/H2-Q1/H2-T3/Gm11127/Trav7d-4/Gm8909/Trav7-4/Cd1d2/Trav14n-2/H2-Bl/H2-Ea/Trav7n-5	12
GO:0042277	peptide binding	14/256	0.000169268	0.025277383	ENSMUSG00000094619/ENSMUSG00000079507/ENSMUSG00000054128/ENSMUSG00000079492/ENSMUSG00000051498/ENSMUSG00000094023/ENSMUSG00000073402/ENSMUSG00000094766/ENSMUSG00000041750/	Trav14d-3-dv8/H2-Q1/H2-T3/Gm11127/Tlr6/Trav7d-4/Gm8909/Trav7-4/Cd1d2/Tr	14

				ENSMUSG00000093801/ ENSMUSG00000073406/ ENSMUSG00000005268/ ENSMUSG00000036322/ ENSMUSG00000105629	av14n-2/H2-Bl/Prlr/H2-Ea/Trav7n-5	
**c) Upregulated pathways within CD8^{+} T cells in C57BL/6 as compared to A/J**						
GO:0002253	Activation of immune response	35/471	4.59796895954495e-07	0.000429335	ENSMUSG00000090272/ ENSMUSG00000073491/ ENSMUSG00000057596/ ENSMUSG00000037849/ ENSMUSG00000021678/ ENSMUSG00000060441/ ENSMUSG00000070501/ ENSMUSG00000050241/ ENSMUSG00000039997/ ENSMUSG00000022899/ ENSMUSG00000066258/ ENSMUSG00000044583/ ENSMUSG00000016024/ ENSMUSG00000052749/ ENSMUSG00000074682/ ENSMUSG00000041827/ ENSMUSG00000037731/ ENSMUSG00000029135/ ENSMUSG00000025314/ ENSMUSG00000078616/ ENSMUSG00000043932/ ENSMUSG00000015854/ ENSMUSG00000052776/ ENSMUSG00000058099/ ENSMUSG00000028885/ ENSMUSG00000046688/ ENSMUSG00000112148/ ENSMUSG00000020641/ ENSMUSG00000029084/ ENSMUSG00000112023/ ENSMUSG00000058715/ ENSMUSG00000109713/ ENSMUSG00000030149/ ENSMUSG00000031639/ ENSMUSG00000024079	Mndal/Ifi213/Trim30d/Ifi206/F2rl1/Trim5/Ifi214/Klre1/Ifi203/Slc15a2/Trim12a/Tlr7/Lbp/Trim30b/Zcchc3/Oasl1/Themis2/Fosl2/Ptprj/Trim30c/Klri2/Cd5l/Oas1a/Nfam1/Smpdl3b/Tifa/Lilrb4a/Rsad2/Cd38/Lilrb4b/Fcer1g/Pvrig/Klrk1/Tlr3/Eif2ak2	35
GO:0050792	Regulation of viral process	17/471	7.64E-06	0.005705842	ENSMUSG00000057596/ ENSMUSG00000060441/ ENSMUSG00000034317/ ENSMUSG00000032690/ ENSMUSG00000056144/ ENSMUSG00000066258/ ENSMUSG00000052749/ ENSMUSG00000041827/ ENSMUSG00000078616/ ENSMUSG00000052776/ ENSMUSG00000009585/ ENSMUSG00000022587/ ENSMUSG00000029561/ ENSMUSG00000020641/ ENSMUSG00000035042/ ENSMUSG00000020262/ ENSMUSG00000024079	Trim30d/Trim5/Trim59/Oas2/Trim34a/Trim12a/Trim30b/Oasl1/Trim30c/Oas1a/Apobec3/Ly6e/Oasl2/Rsad2/Ccl5/Adarb1/Eif2ak2	17

GO:0031349	Positive regulation of defense response	31/471	1.21E-05	0.006470399	ENSMUSG00000090272/ ENSMUSG00000073491/ ENSMUSG00000057596/ ENSMUSG00000037849/ ENSMUSG00000021678/ ENSMUSG00000060441/ ENSMUSG00000050965/ ENSMUSG00000070501/ ENSMUSG00000050241/ ENSMUSG00000039997/ ENSMUSG00000022899/ ENSMUSG00000066258/ ENSMUSG00000044583/ ENSMUSG00000016024/ ENSMUSG00000008734/ ENSMUSG00000052749/ ENSMUSG00000074682/ ENSMUSG00000041827/ ENSMUSG00000078616/ ENSMUSG00000043932/ ENSMUSG00000052776/ ENSMUSG00000059498/ ENSMUSG00000028885/ ENSMUSG00000110195/ ENSMUSG00000046688/ ENSMUSG00000020641/ ENSMUSG00000058715/ ENSMUSG00000030149/ ENSMUSG00000035042/ ENSMUSG00000031639/ ENSMUSG00000024079	Mndal/Ifi213/Trim30d/Ifi206/F2rl1/Trim5/Prkca/Ifi214/Klre1/Ifi203/Slc15a2/Trim12a/Tlr7/Lbp/Gprc5b/Trim30b/Zcchc3/Oasl1/Trim30c/Klri2/Oas1a/Fcgr3/Smpdl3b/Pde2a/Tifa/Rsad2/Fcer1g/Klrk1/Ccl5/Tlr3/Eif2ak2	31
GO:0032602	Chemokine production	12/471	5.98E-05	0.014880085	ENSMUSG00000021678/ ENSMUSG00000044583/ ENSMUSG00000016024/ ENSMUSG00000029135/ ENSMUSG00000020891/ ENSMUSG00000043873/ ENSMUSG00000052776/ ENSMUSG00000112148/ ENSMUSG00000112023/ ENSMUSG00000035042/ ENSMUSG00000031639/ ENSMUSG00000024079	F2rl1/Tlr7/Lbp/Fosl2/Alox8/Chil5/Oas1a/Lilrb4a/Lilrb4b/Ccl5/Tlr3/Eif2ak2	12
GO:0045071	Negative regulation of viral genome replication	8/471	9.48E-05	0.02213604	ENSMUSG00000032690/ ENSMUSG00000041827/ ENSMUSG00000052776/ ENSMUSG00000009585/ ENSMUSG00000029561/ ENSMUSG00000020641/ ENSMUSG00000035042/ ENSMUSG00000024079	Oas2/Oasl1/Oas1a/Apobec3/Oasl2/Rsad2/Ccl5/Eif2ak2	8
GO:0052372	Modulation by symbiont of entry into host	8/471	0.000109964	0.024159841	ENSMUSG00000057596/ ENSMUSG00000060441/ ENSMUSG00000034317/ ENSMUSG00000056144/ ENSMUSG00000066258/ ENSMUSG00000052749/ ENSMUSG00000078616/ ENSMUSG00000022587	Trim30d/Trim5/Trim59/Trim34a/Trim12a/Trim30b/Trim30c/Ly6e	8

GO:0002764	Immune response-regulating signaling pathway	26/471	0.00013542	0.026620775	ENSMUSG00000021678/ENSMUSG00000050241/ENSMUSG00000052013/ENSMUSG00000022899/ENSMUSG00000044583/ENSMUSG00000016024/ENSMUSG00000074682/ENSMUSG00000041827/ENSMUSG00000037731/ENSMUSG00000029135/ENSMUSG00000025314/ENSMUSG00000043932/ENSMUSG00000052776/ENSMUSG00000058099/ENSMUSG00000028885/ENSMUSG00000046688/ENSMUSG00000112148/ENSMUSG00000020641/ENSMUSG00000029084/ENSMUSG00000112023/ENSMUSG00000058715/ENSMUSG00000109713/ENSMUSG00000030149/ENSMUSG00000005672/ENSMUSG00000031639/ENSMUSG00000024079	F2rl1/Klre1/Btla/Slc15a2/Tlr7/Lbp/Zcchc3/Oasl1/Themis2/Fosl2/Ptprj/Klri2/Oas1a/Nfam1/Smpdl3b/Tifa/Lilrb4a/Rsad2/Cd38/Lilrb4b/Fcer1g/Pvrig/Klrk1/Kit/Tlr3/Eif2ak2	26
GO:0031664	Regulation of lipopolysaccharide-mediated signaling pathway	6/471	0.000152016	0.028389079	ENSMUSG00000057596/ENSMUSG00000060441/ENSMUSG00000050965/ENSMUSG00000066258/ENSMUSG00000052749/ENSMUSG00000078616	Trim30d/Trim5/Prkca/Trim12a/Trim30b/Trim30c	6
GO:0007249	Canonical NF-kappaB signal transduction	18/471	0.000185475	0.029123059	ENSMUSG00000057596/ENSMUSG00000021678/ENSMUSG00000060441/ENSMUSG00000034317/ENSMUSG00000056144/ENSMUSG00000066258/ENSMUSG00000044583/ENSMUSG00000008734/ENSMUSG00000052749/ENSMUSG00000078616/ENSMUSG00000095105/ENSMUSG00000000817/ENSMUSG00000046688/ENSMUSG00000112148/ENSMUSG00000031390/ENSMUSG00000112023/ENSMUSG00000068037/ENSMUSG00000031639	Trim30d/F2rl1/Trim5/Trim59/Trim34a/Trim12a/Tlr7/Gprc5b/Trim30b/Trim30c/Edaradd/Fasl/Tifa/Lilrb4a/Avpr2/Lilrb4b/Mas1/Tlr3	18

GO:0051607	Defense response to virus	21/471	0.000187136	0.029123059	ENSMUSG00000035208/ENSMUSG00000057596/ENSMUSG00000021678/ENSMUSG00000060441/ENSMUSG00000030789/ENSMUSG00000032690/ENSMUSG00000056144/ENSMUSG00000066258/ENSMUSG00000044583/ENSMUSG00000052749/ENSMUSG00000074682/ENSMUSG00000041827/ENSMUSG00000078616/ENSMUSG00000052776/ENSMUSG00000009585/ENSMUSG00000029561/ENSMUSG00000020641/ENSMUSG00000035042/ENSMUSG00000022865/ENSMUSG00000031639/ENSMUSG00000024079	Slfn8/Trim30d/F2rl1/Trim5/Itgax/Oas2/Trim34a/Trim12a/Tlr7/Trim30b/Zcchc3/Oasl1/Trim30c/Oas1a/Apobec3/Oasl2/Rsad2/Ccl5/Cxadr/Tlr3/Eif2ak2	21
GO:0035458	Cellular response to interferon-beta	8/471	0.000248609	0.034103231	ENSMUSG00000090272/ENSMUSG00000073491/ENSMUSG00000037849/ENSMUSG00000070501/ENSMUSG00000039997/ENSMUSG00000104713/ENSMUSG00000024617/ENSMUSG00000052776	Mndal/Ifi213/Ifi206/Ifi214/Ifi203/Gbp6/Camk2a/Oas1a	8
GO:0051701	Biological process involved in interaction with host	13/471	0.000264791	0.034103231	ENSMUSG00000057596/ENSMUSG00000060441/ENSMUSG00000020689/ENSMUSG00000071203/ENSMUSG00000034317/ENSMUSG00000056144/ENSMUSG00000066258/ENSMUSG00000104713/ENSMUSG00000025810/ENSMUSG00000052749/ENSMUSG00000097392/ENSMUSG00000078616/ENSMUSG00000022587	Trim30d/Trim5/Itgb3/Naip5/Trim59/Trim34a/Trim12a/Gbp6/Nrp1/Trim30b/Thoc2l/Trim30c/Ly6e	13
GO:0038187	Pattern recognition receptor activity	7/477	3.45E-05	0.01546557	ENSMUSG00000057596/ENSMUSG00000060441/ENSMUSG00000066258/ENSMUSG00000044583/ENSMUSG00000052749/ENSMUSG00000078616/ENSMUSG00000031639	Trim30d/Trim5/Trim12a/Tlr7/Trim30b/Trim30c/Tlr3	7
GO:0030547	Signaling receptor inhibitor activity	6/477	5.31E-05	0.01546557	ENSMUSG00000044177/ENSMUSG00000022587/ENSMUSG00000112148/ENSMUSG00000112023/ENSMUSG00000035042/ENSMUSG00000022584	Wfikkn2/Ly6e/Lilrb4a/Lilrb4b/Ccl5/Ly6c2	6

d) **Downregulated pathways within CD8$^+$ T cells in C57BL/6 as compared to A/J**

GO:0001916	Positive regulation of T cell mediated cytotoxicity	9/269	2.42E-08	4.01E-05	ENSMUSG00000029468/ENSMUSG00000079507/ENSMUSG00000079492/ENSMUSG00000073402/ENSMUSG00000054128/ENSMUSG00000041750/ENSMUSG00000075297/ENSMUSG00000073406/ENSMUSG00000036322	P2rx7/H2-Q1/Gm11127/Gm8909/H2-T3/Cd1d2/H60b/H2-Bl/H2-Ea	9

GO:0002711	Positive regulation of T cell mediated immunity	11/269	2.71E-08	4.01E-05	ENSMUSG00000029468/ ENSMUSG00000079507/ ENSMUSG00000079492/ ENSMUSG00000073402/ ENSMUSG00000054128/ ENSMUSG00000015316/ ENSMUSG00000041750/ ENSMUSG00000075297/ ENSMUSG00000073406/ ENSMUSG00000032691/ ENSMUSG00000036322	P2rx7/H2-Q1/Gm11127/Gm8909/H2-T3/Slamf1/Cd1d2/H60b/H2-Bl/Nlrp3/H2-Ea	11
GO:0002475	Antigen processing and presentation via MHC class Ib	7/269	1.17E-06	0.00043451	ENSMUSG00000079507/ ENSMUSG00000079492/ ENSMUSG00000073402/ ENSMUSG00000054128/ ENSMUSG00000041750/ ENSMUSG00000075297/ ENSMUSG00000073406	H2-Q1/Gm11127/Gm8909/H2-T3/Cd1d2/H60b/H2-Bl	7
GO:0031343	Positive regulation of cell killing	9/269	1.62E-05	0.00220788	ENSMUSG00000029468/ ENSMUSG00000079507/ ENSMUSG00000079492/ ENSMUSG00000073402/ ENSMUSG00000054128/ ENSMUSG00000041750/ ENSMUSG00000075297/ ENSMUSG00000073406/ ENSMUSG00000036322	P2rx7/H2-Q1/Gm11127/Gm8909/H2-T3/Cd1d2/H60b/H2-Bl/H2-Ea	9
GO:0019882	Antigen processing and presentation	10/269	1.64E-05	0.00220788	ENSMUSG00000079507/ ENSMUSG00000019478/ ENSMUSG00000079492/ ENSMUSG00000073402/ ENSMUSG00000054128/ ENSMUSG00000024334/ ENSMUSG00000041750/ ENSMUSG00000075297/ ENSMUSG00000073406/ ENSMUSG00000036322	H2-Q1/Rab4a/Gm11127/Gm8909/H2-T3/H2-Oa/Cd1d2/H60b/H2-Bl/H2-Ea	10
GO:0002438	Acute inflammatory response to antigenic stimulus	6/269	4.87E-05	0.00497139	ENSMUSG00000079507/ ENSMUSG00000054128/ ENSMUSG00000036587/ ENSMUSG00000044288/ ENSMUSG00000026180/ ENSMUSG00000073406	H2-Q1/H2-T3/Fut7/Cnr1/Cxcr2/H2-Bl	6
GO:0002819	Regulation of adaptive immune response	13/269	8.06E-05	0.00746413	ENSMUSG00000029468/ ENSMUSG00000017697/ ENSMUSG00000079507/ ENSMUSG00000079492/ ENSMUSG00000073402/ ENSMUSG00000054128/ ENSMUSG00000015316/ ENSMUSG00000036587/ ENSMUSG00000041750/ ENSMUSG00000075297/ ENSMUSG00000073406/ ENSMUSG00000032691/ ENSMUSG00000036322	P2rx7/Ada/H2-Q1/Gm11127/Gm8909/H2-T3/Slamf1/Fut7/Cd1d2/H60b/H2-Bl/Nlrp3/H2-Ea	13
GO:0032736	Positive regulation of interleukin-13 production	5/269	0.00014303	0.01176856	ENSMUSG00000079507/ ENSMUSG00000039005/ ENSMUSG00000054128/ ENSMUSG00000073406/ ENSMUSG00000032691	H2-Q1/Tlr4/H2-T3/H2-Bl/Nlrp3	5

GO:0002720	Positive regulation of cytokine production involved in immune response	8/269	0.00024221	0.0174979	ENSMUSG00000029468/ ENSMUSG00000079507/ ENSMUSG00000039005/ ENSMUSG00000054128/ ENSMUSG00000015316/ ENSMUSG00000073406/ ENSMUSG00000032691/ ENSMUSG00000038264	P2rx7/H2-Q1/Tlr4/H2-T3/Slamf1/H2-Bl/Nlrp3/Sema7a	8
GO:0046634	Regulation of alpha-beta T cell activation	9/269	0.00038204	0.02648812	ENSMUSG00000017697/ ENSMUSG00000079507/ ENSMUSG00000054128/ ENSMUSG00000041750/ ENSMUSG00000073406/ ENSMUSG00000032691/ ENSMUSG00000027223/ ENSMUSG00000036322/ ENSMUSG00000044813	Ada/H2-Q1/H2-T3/Cd1d2/H2-Bl/Nlrp3/Mapk8ip1/H2-Ea/Shb	9
GO:0002697	Regulation of immune effector process	17/269	0.00038453	0.02648812	ENSMUSG00000029468/ ENSMUSG00000079507/ ENSMUSG00000053158/ ENSMUSG00000079492/ ENSMUSG00000073402/ ENSMUSG00000039005/ ENSMUSG00000054128/ ENSMUSG00000030154/ ENSMUSG00000015316/ ENSMUSG00000036587/ ENSMUSG00000041750/ ENSMUSG00000075297/ ENSMUSG00000073406/ ENSMUSG00000032691/ ENSMUSG00000036322/ ENSMUSG00000044813/ ENSMUSG00000038264	P2rx7/H2-Q1/Fes/Gm11127/Gm8909/Tlr4/H2-T3/Klrb1f/Slamf1/Fut7/Cd1d2/H60b/H2-Bl/Nlrp3/H2-Ea/Shb/Sema7a	17
GO:0032753	Positive regulation of interleukin-4 production	5/269	0.00057435	0.03780526	ENSMUSG00000079507/ ENSMUSG00000054128/ ENSMUSG00000041750/ ENSMUSG00000073406/ ENSMUSG00000032691	H2-Q1/H2-T3/Cd1d2/H2-Bl/Nlrp3	5
GO:2000564	Regulation of CD8-positive, alpha-beta T cell proliferation	4/269	0.00068457	0.04408057	ENSMUSG00000079507/ ENSMUSG00000054128/ ENSMUSG00000073406/ ENSMUSG00000027223	H2-Q1/H2-T3/H2-Bl/Mapk8ip1	4
GO:0042611	MHC protein complex	5/274	0.0001811	0.02906626	ENSMUSG00000079507/ ENSMUSG00000054128/ ENSMUSG00000024334/ ENSMUSG00000073406/ ENSMUSG00000036322	H2-Q1/H2-T3/H2-Oa/H2-Bl/H2-Ea	5
GO:0042605	Peptide antigen binding	12/273	1.95E-10	9.30E-08	ENSMUSG00000079507/ ENSMUSG00000079492/ ENSMUSG00000073402/ ENSMUSG00000054128/ ENSMUSG00000094619/ ENSMUSG00000024334/ ENSMUSG00000094023/ ENSMUSG00000096908/ ENSMUSG00000094766/ ENSMUSG00000104620/ ENSMUSG00000073406/ ENSMUSG00000036322	H2-Q1/Gm11127/Gm8909/H2-T3/Trav14d-3-dv8/H2-Oa/Trav7d-4/Trav7-3/Trav7-4/Trav7-1/H2-Bl/H2-Ea	12

GO:0003823	Antigen binding	13/273	1.46E-08	3.48E-06	ENSMUSG00000079507/ ENSMUSG00000079492/ ENSMUSG00000073402/ ENSMUSG00000054128/ ENSMUSG00000094619/ ENSMUSG00000024334/ ENSMUSG00000094023/ ENSMUSG00000096908/ ENSMUSG00000041750/ ENSMUSG00000094766/ ENSMUSG00000104620/ ENSMUSG00000073406/ ENSMUSG00000036322	H2- Q1/Gm111 27/Gm8909 /H2- T3/Trav14d -3-dv8/H2- Oa/Trav7d- 4/Trav7- 3/Cd1d2/Tr av7- 4/Trav7- 1/H2- Bl/H2-Ea	13
GO:0042277	Peptide binding	14/273	0.0002332 5	0.0277562	ENSMUSG00000079507/ ENSMUSG00000079492/ ENSMUSG00000073402/ ENSMUSG00000051498/ ENSMUSG00000054128/ ENSMUSG00000094619/ ENSMUSG00000024334/ ENSMUSG00000094023/ ENSMUSG00000096908/ ENSMUSG00000041750/ ENSMUSG00000094766/ ENSMUSG00000104620/ ENSMUSG00000073406/ ENSMUSG00000036322	H2- Q1/Gm111 27/Gm8909 /Tlr6/H2- T3/Trav14d -3-dv8/H2- Oa/Trav7d- 4/Trav7- 3/Cd1d2/Tr av7- 4/Trav7- 1/H2- Bl/H2-Ea	14

Supplementary Table S6. The list of genes contributing to various pathways revealed by KEGG enrichment analysis

KEGGID	Description	Gene Ratio	P value	Padj	Gene ID	Gene Name	Count
**a) Downregulated genes within CD4$^+$ T cells in C57BL/6 as compared to A/J**							
mmu05330	Allograft rejection	9/184	4.60E-06	0.00058421	ENSMUSG00000079507/ ENSMUSG00000054128/ ENSMUSG00000079492/ ENSMUSG00000073402/ ENSMUSG00000073403/ ENSMUSG00000015437/ ENSMUSG00000092457/ ENSMUSG00000073406/ ENSMUSG00000036322	H2-Q1/H2-T3/Gm11127/Gm8909/Gm10499/Gzmb/Gm8835/H2-Bl/H2-Ea	9
mmu05332	Graft-versus-host disease	9/184	5.25E-06	0.00058421	ENSMUSG00000079507/ ENSMUSG00000054128/ ENSMUSG00000079492/ ENSMUSG00000073402/ ENSMUSG00000073403/ ENSMUSG00000015437/ ENSMUSG00000092457/ ENSMUSG00000073406/ ENSMUSG00000036322	H2-Q1/H2-T3/Gm11127/Gm8909/Gm10499/Gzmb/Gm8835/H2-Bl/H2-Ea	9
mmu05320	Autoimmune thyroid disease	9/184	7.69E-06	0.00058421	ENSMUSG00000079507/ ENSMUSG00000054128/ ENSMUSG00000079492/ ENSMUSG00000073402/ ENSMUSG00000073403/ ENSMUSG00000015437/ ENSMUSG00000092457/ ENSMUSG00000073406/ ENSMUSG00000036322	H2-Q1/H2-T3/Gm11127/Gm8909/Gm10499/Gzmb/Gm8835/H2-Bl/H2-Ea	9
mmu04940	Type I diabetes mellitus	9/184	1.74E-05	0.0009898	ENSMUSG00000079507/ ENSMUSG00000054128/ ENSMUSG00000079492/ ENSMUSG00000073402/ ENSMUSG00000073403/ ENSMUSG00000015437/ ENSMUSG00000092457/ ENSMUSG00000073406/ ENSMUSG00000036322	H2-Q1/H2-T3/Gm11127/Gm8909/Gm10499/Gzmb/Gm8835/H2-Bl/H2-Ea	9
mmu05165	Human papillomavirus infection	20/184	9.67E-05	0.00372425	ENSMUSG00000112003/ ENSMUSG00000079575/ ENSMUSG00000079507/ ENSMUSG00000080242/ ENSMUSG00000030170/ ENSMUSG00000054128/ ENSMUSG00000061062/ ENSMUSG00000079492/ ENSMUSG00000039347/ ENSMUSG00000025348/ ENSMUSG00000073402/ ENSMUSG00000032572/ ENSMUSG00000015829/ ENSMUSG00000000125/ ENSMUSG00000073403/ ENSMUSG00000048001/ ENSMUSG00000081121/ ENSMUSG00000092457/ ENSMUSG00000073406/ ENSMUSG00000026167	Gm4864/Rbpj-ps3/H2-Q1/Atp6v0c-ps2/Wnt5b/H2-T3/Hdac1-ps/Gm11127/Atp6v0e2/Itga7/Gm8909/Col6a4/Tnr/Wnt3/Gm10499/Hes5/Gm12791/Gm8835/H2-Bl/Wnt10a	20

mmu04612	Antigen processing and presentation	10/184	0.00010706	0.00372425	ENSMUSG00000079507/ ENSMUSG00000054128/ ENSMUSG00000079492/ ENSMUSG00000100801/ ENSMUSG00000073402/ ENSMUSG00000073403/ ENSMUSG00000059970/ ENSMUSG00000092457/ ENSMUSG00000073406/ ENSMUSG00000036322	H2-Q1/H2-T3/Gm11127/Gm15459/Gm8909/Gm10499/Hspa2/Gm8835/H2-Bl/H2-Ea	10
mmu04145	Phagosome	14/184	0.00011434	0.00372425	ENSMUSG00000079507/ ENSMUSG00000080242/ ENSMUSG00000054128/ ENSMUSG00000079492/ ENSMUSG00000051498/ ENSMUSG00000039347/ ENSMUSG00000073402/ ENSMUSG00000073403/ ENSMUSG00000039005/ ENSMUSG00000082809/ ENSMUSG00000092457/ ENSMUSG00000073406/ ENSMUSG00000114547/ ENSMUSG00000036322	H2-Q1/Atp6v0c-ps2/H2-T3/Gm11127/Tlr6/Atp6v0e2/Gm8909/Gm10499/Tlr4/Gm14150/Gm8835/H2-Bl/Gm3226/H2-Ea	14
mmu05416	Viral myocarditis	8/184	0.00152312	0.04340902	ENSMUSG00000079507/ ENSMUSG00000054128/ ENSMUSG00000079492/ ENSMUSG00000073402/ ENSMUSG00000073403/ ENSMUSG00000092457/ ENSMUSG00000073406/ ENSMUSG00000036322	H2-Q1/H2-T3/Gm11127/Gm8909/Gm10499/Gm8835/H2-Bl/H2-Ea	8

b) Upregulated genes within CD8^{+} T cells in C57BL/6 as compared to A/J

mmu04650	Natural killer cell mediated cytotoxicity	12/235	3.33E-05	0.00889574	ENSMUSG00000050965/ ENSMUSG00000079853/ ENSMUSG00000067591/ ENSMUSG00000067599/ ENSMUSG00000089727/ ENSMUSG00000033024/ ENSMUSG00000000817/ ENSMUSG00000015437/ ENSMUSG00000058715/ ENSMUSG00000030149/ ENSMUSG00000030774/ ENSMUSG00000072721	Prkca/Klra1/Klra3/Klra7/Klra8/Klra9/Fasl/Gzmb/Fcer1g/Klrk1/Pak1/Klra14-ps	12
mmu05168	Herpes simplex virus 1 infection	27/235	0.00018792	0.02508738	ENSMUSG00000020689/ ENSMUSG00000074733/ ENSMUSG00000032690/ ENSMUSG00000058402/ ENSMUSG00000066009/ ENSMUSG00000058900/ ENSMUSG00000098900/ ENSMUSG00000079505/ ENSMUSG00000066647/ ENSMUSG00000069206/ ENSMUSG00000068130/ ENSMUSG00000068959/ ENSMUSG00000000817/ ENSMUSG00000030424/ ENSMUSG00000053985/ ENSMUSG00000060336/ ENSMUSG00000052776/ ENSMUSG00000030823/ ENSMUSG00000112640/ ENSMUSG00000100235/ ENSMUSG00000106161/	Itgb3/Zfp950/Oas2/Zfp420/Zfp987/Rsl1/Gm18190/Gm11131/Gm5113/Zfp874a/Zfp442/Zfp619/Fasl/Zfp939/Zfp14/Zfp937/Oas1a/9130019O22Rik/Gm32687/Gm28557/Pvrig-ps/BC051537/Itga5/Ccl5/Tlr3/Zfp82/Eif2ak2	27

					ENSMUSG00000098197/ENSMUSG00000000555/ENSMUSG00000035042/ENSMUSG00000031639/ENSMUSG00000098022/ENSMUSG00000024079		
mmu00140	Steroid hormone biosynthesis	5/235	0.00064361	0.04052744	ENSMUSG00000109602/ENSMUSG00000003271/ENSMUSG00000061740/ENSMUSG00000003555/ENSMUSG00000021594	Gm31597/Sult2b1/Cyp2d22/Cyp17a1/Srd5a1	5
mmu04640	Hematopoietic cell lineage	9/235	0.00082523	0.04052744	ENSMUSG00000020689/ENSMUSG00000005087/ENSMUSG00000042284/ENSMUSG00000059326/ENSMUSG00000025014/ENSMUSG00000029084/ENSMUSG00000098197/ENSMUSG00000000555/ENSMUSG00000005672	Itgb3/Cd44/Itga1/Csf2ra/Dntt/Cd38/BC051537/Itga5/Kit	9
mmu00531	Glycosaminoglycan degradation	4/235	0.00097047	0.04052744	ENSMUSG00000033540/ENSMUSG00000037260/ENSMUSG00000042082/ENSMUSG00000113647	Idua/Hgsnat/Arsb/Gm47210	4
mmu05143	African trypanosomiasis	5/235	0.00104521	0.04052744	ENSMUSG00000021678/ENSMUSG00000050965/ENSMUSG00000000817/ENSMUSG00000039943/ENSMUSG00000024639	F2rl1/Prkca/Fasl/Plcb4/Gnaq	5
mmu04916	Melanogenesis	8/235	0.00106252	0.04052744	ENSMUSG00000050965/ENSMUSG00000038648/ENSMUSG00000024617/ENSMUSG00000057897/ENSMUSG00000039943/ENSMUSG00000024639/ENSMUSG00000035158/ENSMUSG00000005672	Prkca/Creb3l2/Camk2a/Camk2b/Plcb4/Gnaq/Mitf/Kit	8

c) Downregulated genes within CD8+ T cells in C57BL/6 as compared to A/J

mmu05165	Human papillomavirus infection	27/190	2.36E-08	5.69E-06	ENSMUSG00000112003/ENSMUSG00000080242/ENSMUSG00000073403/ENSMUSG00000079507/ENSMUSG00000030170/ENSMUSG00000079575/ENSMUSG00000079492/ENSMUSG00000061062/ENSMUSG00000027111/ENSMUSG00000073402/ENSMUSG00000032572/ENSMUSG00000015829/ENSMUSG00000054128/ENSMUSG00000092457/ENSMUSG00000031502/ENSMUSG00000025348/ENSMUSG00000084347/ENSMUSG00000038146/ENSMUSG00000048001/ENSMUSG00000073406/ENSMUSG00000116544/ENSMUSG00000039347/ENSMUSG00000045007/ENSMUSG00000023341/ENSMUSG00000026167/ENSMUSG00000045095/ENSMUSG00000109841	Gm4864/Atp6v0c-ps2/Gm10499/H2-Q1/Wnt5b/Rbpj-ps3/Gm11127/Hdac1-ps/Itga6/Gm8909/Col6a4/Tnr/H2-T3/Gm8835/Col4a1/Itga7/Akt2-ps/Notch3/Hes5/H2-Bl/Gm49626/Atp6v0e2/Tubg2/Mx2/Wnt10a/Magi1/E330011O21Rik	27

mmu04612	Antigen processing and presentation	12/190	6.08E-06	0.00053336	ENSMUSG00000073403/ENSMUSG00000079507/ENSMUSG00000079492/ENSMUSG00000100801/ENSMUSG00000073402/ENSMUSG00000054128/ENSMUSG00000024334/ENSMUSG00000092457/ENSMUSG00000073406/ENSMUSG00000083899/ENSMUSG00000059970/ENSMUSG00000036322	Gm10499/H2-Q1/Gm11127/Gm15459/Gm8909/H2-T3/H2-Oa/Gm8835/H2-Bl/Gm12346/Hspa2/H2-Ea	12
mmu05330	Allograft rejection	9/190	8.85E-06	0.00053336	ENSMUSG00000073403/ENSMUSG00000079507/ENSMUSG00000079492/ENSMUSG00000073402/ENSMUSG00000054128/ENSMUSG00000024334/ENSMUSG00000092457/ENSMUSG00000073406/ENSMUSG00000036322	Gm10499/H2-Q1/Gm11127/Gm8909/H2-T3/H2-Oa/Gm8835/H2-Bl/H2-Ea	9
mmu05332	Graft-versus-host disease	9/190	8.85E-06	0.00053336	ENSMUSG00000073403/ENSMUSG00000079507/ENSMUSG00000079492/ENSMUSG00000073402/ENSMUSG00000054128/ENSMUSG00000024334/ENSMUSG00000092457/ENSMUSG00000073406/ENSMUSG00000036322	Gm10499/H2-Q1/Gm11127/Gm8909/H2-T3/H2-Oa/Gm8835/H2-Bl/H2-Ea	9
mmu05320	Autoimmune thyroid disease	9/190	1.81E-05	0.00087048	ENSMUSG00000073403/ENSMUSG00000079507/ENSMUSG00000079492/ENSMUSG00000073402/ENSMUSG00000054128/ENSMUSG00000024334/ENSMUSG00000092457/ENSMUSG00000073406/ENSMUSG00000036322	Gm10499/H2-Q1/Gm11127/Gm8909/H2-T3/H2-Oa/Gm8835/H2-Bl/H2-Ea	9
mmu04940	Type I diabetes mellitus	9/190	2.79E-05	0.00112245	ENSMUSG00000073403/ENSMUSG00000079507/ENSMUSG00000079492/ENSMUSG00000073402/ENSMUSG00000054128/ENSMUSG00000024334/ENSMUSG00000092457/ENSMUSG00000073406/ENSMUSG00000036322	Gm10499/H2-Q1/Gm11127/Gm8909/H2-T3/H2-Oa/Gm8835/H2-Bl/H2-Ea	9
mmu04145	Phagosome	14/190	0.00016183	0.00557168	ENSMUSG00000080242/ENSMUSG00000073403/ENSMUSG00000079507/ENSMUSG00000079492/ENSMUSG00000073402/ENSMUSG00000051498/ENSMUSG00000039005/ENSMUSG00000054128/ENSMUSG00000024334/ENSMUSG00000114547/ENSMUSG00000092457/ENSMUSG00000073406/ENSMUSG00000039347/ENSMUSG00000036322	Atp6v0c-ps2/Gm10499/H2-Q1/Gm11127/Gm8909/Tlr6/Tlr4/H2-T3/H2-Oa/Gm3226/Gm8835/H2-Bl/Atp6v0e2/H2-Ea	14

mmu05169	Epstein-Barr virus infection	16/190	0.00027658	0.00833198	ENSMUSG00000112003/ ENSMUSG00000073403/ ENSMUSG00000079507/ ENSMUSG00000079575/ ENSMUSG00000079492/ ENSMUSG00000061062/ ENSMUSG00000073402/ ENSMUSG00000054128/ ENSMUSG00000024334/ ENSMUSG00000092457/ ENSMUSG00000084347/ ENSMUSG00000112163/ ENSMUSG00000073406/ ENSMUSG00000116544/ ENSMUSG00000036322/ ENSMUSG00000109841	Gm4864/Gm10499/H2-Q1/Rbpj-ps3/Gm11127/Hdac1-ps/Gm8909/H2-T3/H2-Oa/Gm8835/Akt2-ps/Gm8188/H2-Bl/Gm49626/H2-Ea/E330011O21Rik	16
mmu04514	Cell adhesion molecules	11/190	0.00034654	0.00927948	ENSMUSG00000073403/ ENSMUSG00000079507/ ENSMUSG00000079492/ ENSMUSG00000027111/ ENSMUSG00000073402/ ENSMUSG00000054128/ ENSMUSG00000024334/ ENSMUSG00000033295/ ENSMUSG00000092457/ ENSMUSG00000073406/ ENSMUSG00000036322	Gm10499/H2-Q1/Gm11127/Itga6/Gm8909/H2-T3/H2-Oa/Ptprf/Gm8835/H2-Bl/H2-Ea	11
mmu05416	Viral myocarditis	9/190	0.00061998	0.01494149	ENSMUSG00000073403/ ENSMUSG00000079507/ ENSMUSG00000079492/ ENSMUSG00000073402/ ENSMUSG00000054128/ ENSMUSG00000024334/ ENSMUSG00000092457/ ENSMUSG00000073406/ ENSMUSG00000036322	Gm10499/H2-Q1/Gm11127/Gm8909/H2-T3/H2-Oa/Gm8835/H2-Bl/H2-Ea	9
mmu05203	Viral carcinogenesis	14/190	0.00177273	0.03883892	ENSMUSG00000112003/ ENSMUSG00000073403/ ENSMUSG00000079507/ ENSMUSG00000079575/ ENSMUSG00000079492/ ENSMUSG00000061062/ ENSMUSG00000073402/ ENSMUSG00000054128/ ENSMUSG00000092457/ ENSMUSG00000118266/ ENSMUSG00000073406/ ENSMUSG00000069305/ ENSMUSG00000060981/ ENSMUSG00000109841	Gm4864/Gm10499/H2-Q1/Rbpj-ps3/Gm11127/Hdac1-ps/Gm8909/H2-T3/Gm8835/Gm18371/H2-Bl/H4c18/H4c8/E330011O21Rik	14

Supplementary Table S7. Selected the top 30 transcription factors linked to the transcriptomic profiles of $CD4^+$ and $CD8^+$ transgenic T cells of C57Bl/6 in relation to A/J mice

Cell type	TFs associated with upregulated genes	TFs associated with downregulated genes
Patterns common to both $CD4^+$ and $CD8^+$ T cells	ESR1<br>CREBBP<br>AR<br>ZFP263<br>TRP3<br>STAT3<br>SMAD3<br>RELA<br>NFKB1<br>GATA3	EGR1<br>E2F1<br>CEBPB<br>STAT1<br>MYC<br>JUN<br>IRF4<br>FOS<br>ETS1
Pattern unique to $CD4^+$ T cells	E2F4<br>CEBPA<br>TBX21<br>HIF1a	ZBTB7a<br>SP3<br>SMAD4<br>POU2F2<br>MAZ<br>SP1<br>RUNX2
Pattern unique to $CD8^+$ T cells	TCF4<br>RUNX1<br>NFE2L2<br>SP1<br>RUNX2	BHLHE40<br>ATF3<br>TCF3<br>SREBF1<br>ID2<br>HIF1a

Supplementary Table S8. Associations between transcription factors and the DEGs in the CD4$^+$ and CD8$^+$ T cell subsets

Upregulated genes	Downregulated genes	Transcription factors
DEGs of CD4$^+$ T cells linked to transcription factor		
*	*	TBX21
*	*	POU2F2
TNC	IFI204	SMAD4
DAPK1	*	
FGF2	*	E2F4
LBP	CXCR2	CEBPA
*	ALPL	SP3
EPB41L1	*	MAZ
*	MXRA8	ZBTB7A
DEGs of CD8$^+$ T cells linked to transcription factor		
*	*	ATF3
*	*	BHLHE40
*	*	TCF3
*	*	TCF4
CD5L	SCD1	SREBF1
ITGAX	*	RUNX1
*	SLC7A	NFE2L2
*	ALPL	ID2

*No genes relevant to the indicated transcription factors were noted in either up- or downregulated categories in the respective subset.
